# Elaiophylin triggers paraptosis and preferentially kills ovarian cancer drug-resistant cells by inducing MAPK hyperactivation

**DOI:** 10.1038/s41392-022-01131-7

**Published:** 2022-09-12

**Authors:** Guan-Nan Li, Xue-Jiao Zhao, Zhen Wang, Meng-Shi Luo, Shen-Nan Shi, Dan-Mei Yan, Hua-Yi Li, Jia-Hao Liu, Yang Yang, Jia-Hong Tan, Ze-Yu Zhang, Ru-Qi Chen, Hui-Ling Lai, Xiao-Yuan Huang, Jian-Feng Zhou, Ding Ma, Yong Fang, Qing-Lei Gao

**Affiliations:** 1 Cancer Biology Research Center (Key Laboratory of the Ministry of Education), Tongji Hospital, Tongji Medical College, Huazhong University of Science and Technology, 430030 Wuhan, Hubei China; 2 National Clinical Research Center for Obstetrics and Gynecology, Department of Gynecological Oncology, Tongji Hospital, Tongji Medical College, Huazhong University of Science and Technology, 430030 Wuhan, Hubei China; 3 Department of Hematology, Tongji Hospital, Tongji Medical College, Huazhong University of Science and Technology, 430030 Wuhan, Hubei China; 4 Department of Gynecology, the Sixth Affiliated Hospital, Sun Yat-Sen University, 510000 Guangzhou, Guangdong China

**Keywords:** Gynaecological cancer, Target identification, Drug development, Molecular medicine

## Abstract

Finely tuned mitogen-activated protein kinase (MAPK) signaling is important for cancer cell survival. Perturbations that push cells out of the MAPK fitness zone result in cell death. Previously, in a screen of the North China Pharmaceutical Group Corporation’s pure compound library of microbial origin, we identified elaiophylin as an autophagy inhibitor. Here, we demonstrated a new role for elaiophylin in inducing excessive endoplasmic reticulum (ER) stress, ER-derived cytoplasmic vacuolization, and consequent paraptosis by hyperactivating the MAPK pathway in multiple cancer cells. Genome-wide CRISPR/Cas9 knockout library screening identified SHP2, an upstream intermediary of the MAPK pathway, as a critical target in elaiophylin-induced paraptosis. The cellular thermal shift assay (CETSA) and surface plasmon resonance (SPR) assay further confirmed the direct binding between the SHP2 and elaiophylin. Inhibition of the SHP2/SOS1/MAPK pathway through SHP2 knockdown or pharmacological inhibitors distinctly attenuated elaiophylin-induced paraptosis and autophagy inhibition. Interestingly, elaiophylin markedly increased the already-elevated MAPK levels and preferentially killed drug-resistant cells with enhanced basal MAPK levels. Elaiophylin overcame drug resistance by triggering paraptosis in multiple tumor-bearing mouse models resistant to platinum, taxane, or PARPi, suggesting that elaiophylin might offer a reasonable therapeutic strategy for refractory ovarian cancer.

## Introduction

Aberrantly activated mitogen-activated protein kinase (MAPK) signaling exists in over 85% of the cancers and accounts for more than 40% of cancer cases.^1^ MAPK pathway inhibitors are under laboratory and clinical investigation to identify their efficacy against various tumors,^2^ but MAPK signal reactivation could invariably develop and lead to treatment failure.^3^ Interestingly, cancer cells that acquire drug resistance are used to an optimal level of MAPK signaling,^4^ and enhancing MAPK activity in these cells has antitumor effects. For example, acute MAPK inhibitor withdrawal or *ERK2* overexpression in drug-addicted resistant cells results in excessive MAPK signaling, which causes cytotoxicity.^4,5^ Knockout of negative MAPK regulators protein phosphatase 6 catalytic subunit (*PPP6C*) or dual specificity phosphatase 4/6 (*DUSP4/6*) results in MAPK overactivation and selectively impairs the viability of melanoma cells with increased baseline levels of MAPK.^6,7^ Maladjusted MAPK could even reportedly cause apoptosis, slow-cycling, senescence, and parthanatos-related cell death in resistant melanoma cells.^8^ These phenomena indicate that cancer cells require a finely tuned MAPK fitness zone, which will not be tolerated above a certain threshold.

Epithelial ovarian cancer (EOC), the most lethal gynecological cancer, often evades early detection and defies treatment.^9^ As screenings are not recommended in average-risk asymptomatic women, most EOCs (>80%) are diagnosed at an advanced stage.^10^ For the last two decades, debulking surgeries followed by adjuvant platinum/taxane-based chemotherapy have been the standard treatment for EOC, with encouraging clinical response in newly diagnosed patients.^11^ Unfortunately, most EOCs become incurable due to the emergence of chemoresistance.^12^ Recently, poly (ADP-ribose) polymerase inhibitors (PARPi) have revolutionized EOC management.^13^ However, acquired resistance to PARPi is still inevitable.^14,15^ In EOC, MAPK activation has been proven to weaken the efficacy of platinum, taxane, and PARPi.^16–18^ Specifically, microtubule-associated serine/threonine kinase 1 (*MAST1*) induced MAPK activation contributes to platinum insensitivity and is associated with shortened survival in EOC.^16^ Upregulation of the RAF/MEK/ERK pathway downregulates FoxM1 to engender paclitaxel resistance.^17^ Increased RAS/MAPK activities lead to impaired response to PARPi by regulating the FOXO3a-BIM cascade.^18^ Therefore, introducing agents capable of eradicating resistant tumor cells is an essential avenue to improved outcomes of EOC.

Paraptosis is a specific form of programmed cell death (PCD) accompanied by massive cytoplasmic vacuoles involving the dilation of endoplasmic reticulum (ER) and/or mitochondria.^19^ Paraptosis is caspase-independent and lacks the typical morphological changes of apoptosis, but requires transcription and translation. Thus, inhibition of protein synthesis by cycloheximide (CHX) could effectively block paraptosis,^20^ while apoptosis inhibition such as caspase inhibitors shows no affection.^21^ In most cases, paraptosis is mediated by MAPKs, such as MEKs or JNKs. Thus, paraptosis can be rescued by specific inhibition of these kinases, including inhibitors or siRNAs, and can also be specifically blocked by AIP-1/Alix (apoptosis-linkedgene-2-interacting protein-1, also known as Alix).^19^

We hypothesized that by inducing MAPK activation-mediated paraptosis, drug-resistant cancer cells with intrinsically increased MAPK signaling could be preferentially eliminated. Here, we demonstrated that elaiophylin, a natural C_2_-symmetric macrodiolide antibiotic isolated from *Streptomyces melanosporus*,^22^ induced ER-derived vacuolization and consequent paraptosis through the hyperactivation of MAPK pathway. More importantly, MAPK activation in platinum-, taxane-, or PARPi-resistant cancer cells, which endowed them with survival advantages against cytotoxic drugs, simultaneously increased their susceptibility to elaiophylin and became an exploitable therapeutic target for refractory EOC.

## Results

### Elaiophylin induces cytoplasmic vacuolization in ovarian cancer cells

**Fig. 1:**
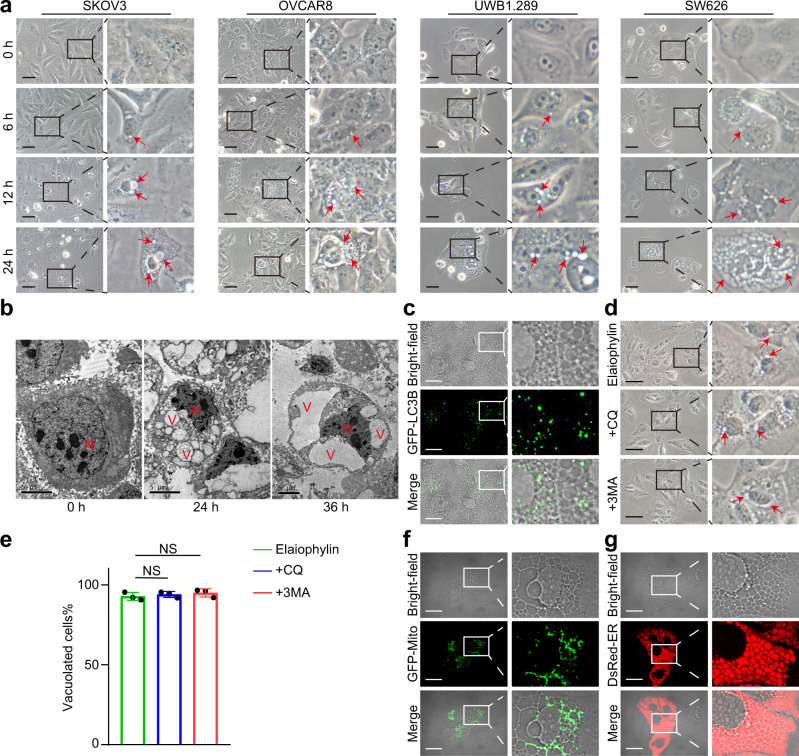
Elaiophylin induces ER-derived vacuoles in ovarian cancer cells. **a** SKOV3, OVCAR8, UWB1.289, and SW626 cells were exposed to 0.5 µM elaiophylin and observed by light microscopy. Arrows indicate cytoplasmic vacuoles. Scale bars: 50 µm. **b** SKOV3 cells were exposed to 0.5 µM elaiophylin and observed by transmission electron microscopy. N indicates cell nucleus. V indicates cytoplasmic vacuoles. Scale bars: 5 µm. **c** SKOV3 cells transfected with GFP-LC3B plasmid were exposed to 0.5 µM elaiophylin for 12 h, and observed under the confocal microscope. Representative bright-field and fluorescence images are shown. Scale bar: 20 µm. **d** SKOV3 cells were exposed to 0.5 µM elaiophylin alone or in combination with CQ (25 µM) or 3-MA (5 mM) for 12 h, and observed by light microscopy. Arrows indicate cytoplasmic vacuoles. Scale bar: 50 µm. **e** The proportion of cells displaying vacuoles in (**d**) was scored by visually examining at least 100 cells. Data are mean ± SD of three independent experiments (Two-tailed unpaired Student’s *t*-test, NS, *p* > 0.05). **f**, **g** SKOV3 cells expressing GFP-Mito (**f**) or DsRed-ER (**g**) were exposed to 0.5 µM elaiophylin for 12 h, and observed under the confocal microscope. Representative bright-field and fluorescence images are shown. Scale bar: 20 µm

Previously, we identified elaiophylin as a late-stage autophagy inhibitor based on functional screening of 540 natural compounds of microbial origin.^23^ Meanwhile, we noticed that, unlike the typical morphological characteristics of apoptosis, the death of elaiophylin-treated ovarian cancer cell lines was preceded by the formation and accumulation of massive cytoplasmic vacuoles in the vicinity of the nucleus. Vacuoles fused and enlarged when moving towards the cell periphery (Fig. 1a). The same phenomena were observed in primary ovarian cancer cells and cell lines of other cancers (A549, MDA-MB-231, SiHa, and SW480) treated with elaiophylin (Supplementary Fig. S1-2). Transmission electron microscopy was used to reveal the ultrastructural characteristics of this process, showing single-membrane vacuoles surrounding intact nuclei in elaiophylin-treated SKOV3 cells (Fig. 1b).

To determine whether the elaiophylin-induced vacuoles were related to autophagy, we used SKOV3 cells expressing GFP-LC3B, and found the vacuoles did not colocalize with GFP-LC3B (Fig. 1c and Supplementary Fig. S3a). Furthermore, the addition of the autophagy inhibitors chloroquine (CQ) or 3-methyladenine (3-MA) was not able to block elaiophylin-induced vacuoles (Fig. 1d, e and Supplementary Fig. S3b, c). To investigate the relevance between elaiophylin-induced cell death and several established forms of cell death, we examined the hallmarks of apoptosis (cleavage of caspase 3 and PARP), pyroptosis (cleavage of GSDMD or GSDME), necroptosis (phosphorylation of RIPK1 and MLKL), and ferroptosis (lipid peroxidation). We only observed the cleavage of caspase 3 and PARP after 12 h of elaiophylin treatment and positive Annexin V/PI staining, indicating that apoptosis was involved in elaiophylin-induced cell death (Supplementary Fig. S4a–e). However, the apoptosis inhibitor Z-VAD-FMK could not block the formation of vacuoles (Supplemental Fig. S4f). In addition, no obvious cell cycle arrest was observed in elaiophylin-treated cells (Supplemental Fig. S4g). To investigate the origin of vacuoles in elaiophylin-treated cells, we transfected them with a fluorescent mitochondrial marker (GFP-Mito) or an ER marker (DsRed-ER). Vacuoles colocalized precisely with DsRed-ER fluorescence rather than GFP-Mito fluorescence (Fig. 1f, g). Taken together, these data demonstrate that elaiophylin-induced vacuoles originate from the ER.

### Elaiophylin induces ER stress and consequent paraptosis in ovarian cancer cells

**Fig. 2:**
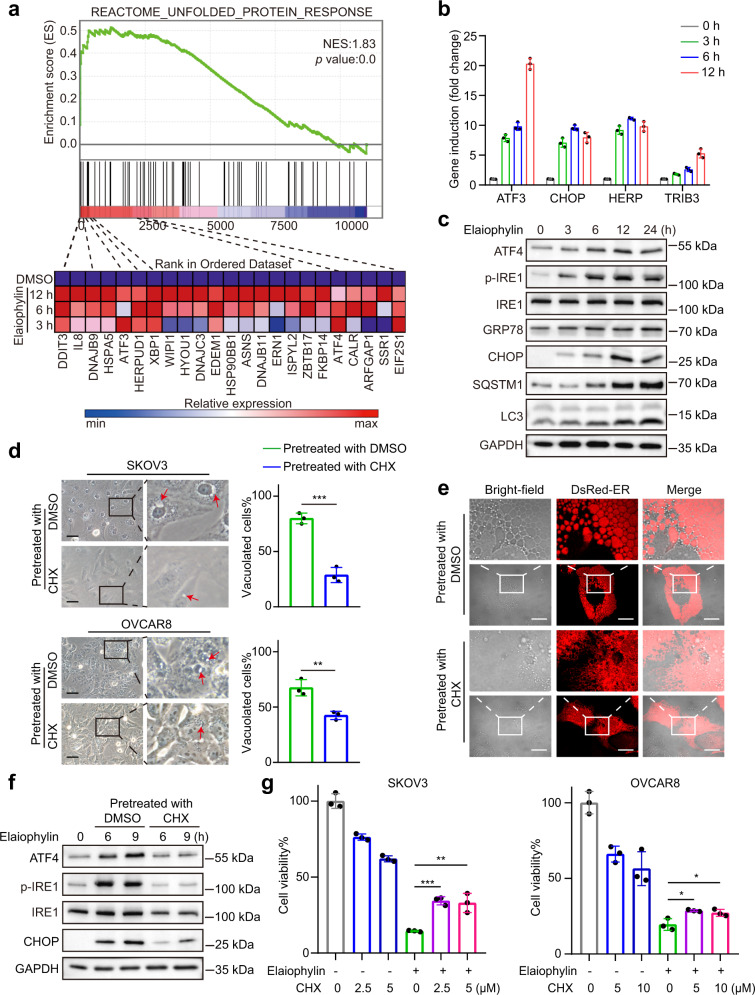
Elaiophylin induces paraptosis in ovarian cancer cells. **a** Top: gene set enrichment analysis of the unfolded protein response in SKOV3 cells exposed to elaiophylin. Bottom: heatmaps of unfolded protein response gene sets in SKOV3 cells exposed to elaiophylin. **b** The mRNA relative expression of indicated genes in SKOV3 cells exposed to 0.5 µM elaiophylin. **c** Assessment of indicated protein levels using western blotting in SKOV3 cells exposed to 0.5 µM elaiophylin. **d** Left panel: light microscopy images of SKOV3 and OVCAR8 cells exposed for 9 h to 0.5 µM elaiophylin with pretreatment of DMSO or CHX (5 µM for 4 h). Arrows indicate cytoplasmic vacuoles. Scale bar: 50 µm. Right panel: proportion of cells displaying vacuoles was scored by visually examining at least 100 cells. Data are mean ± SD of three independent experiments (Two-tailed unpaired Student’s *t* test, **p < 0.01, ***p < 0.001). **e** Representative fluorescence and bright-field images of SKOV3 cells expressing DsRed-ER exposed to 0.5 µM elaiophylin for 9 h with pretreatment of DMSO or CHX (5 µM for 4 h). Scale bar: 20 µm. **f** Assessment of indicated protein levels using western blotting in SKOV3 cells exposed to 0.5 µM elaiophylin with pretreatment of DMSO or CHX (5 µM for 4 h). **g** The viability of SKOV3 and OVCAR8 cells exposed to 0.5 µM elaiophylin for 36 h with pretreatment of DMSO or CHX (4 h). Data are mean ± SD of three independent experiments (Two-tailed unpaired Student’s *t* test, *p < 0.05, **p < 0.01, ***p < 0.001)

We next explored whether elaiophylin-induced ER stress in ovarian cancer cells. Gene set enrichment analysis (GSEA) conducted on the microarray data of SKOV3 cells treated with elaiophylin for 0, 3, 6, or 12 h found a significant enrichment of gene sets involved in unfolded protein response, such as *DDIT3* (CHOP), *ATF3*, and *HERPUD1* (Fig. 2a), which were further confirmed by real-time PCR (Fig. 2b). Besides, elaiophylin caused phosphorylation of IRE1 and upregulated the protein levels of ATF4 and CHOP, suggesting that elaiophylin-induced ER stress in SKOV3 cells (Fig. 2c).

Paraptosis is a nonapoptotic PCD featured by ER stress and extensive cytoplasmic vacuoles originated from the ER. To further investigate whether elaiophylin induces paraptosis, we pretreated SKOV3 and OVCAR8 cells with CHX, which blocks paraptotic vacuoles, and found that CHX pretreatment effectively diminished elaiophylin-induced vacuoles and ER dilatation (Fig. 2d, e). Consistently, CHX pretreatment attenuated elaiophylin-induced upregulation of ATF4, p-IRE1, and CHOP, indicating that elaiophylin-induced ER stress was alleviated (Fig. 2f). Furthermore, CHX pretreatment partially rescued the decrease in cell viability induced by elaiophylin (Fig. 2g and Supplementary Fig. S5). In addition, exogenous overexpression of Alix in SKOV3 cells alleviated ER stress, cytoplasmic vacuolization, and elaiophylin-induced cell death (Supplementary Fig. S6). Altogether, these data indicate that elaiophylin induces paraptotic cell death in ovarian cancer cells.

### Involvement of MAPKs in elaiophylin-induced paraptosis

**Fig. 3:**
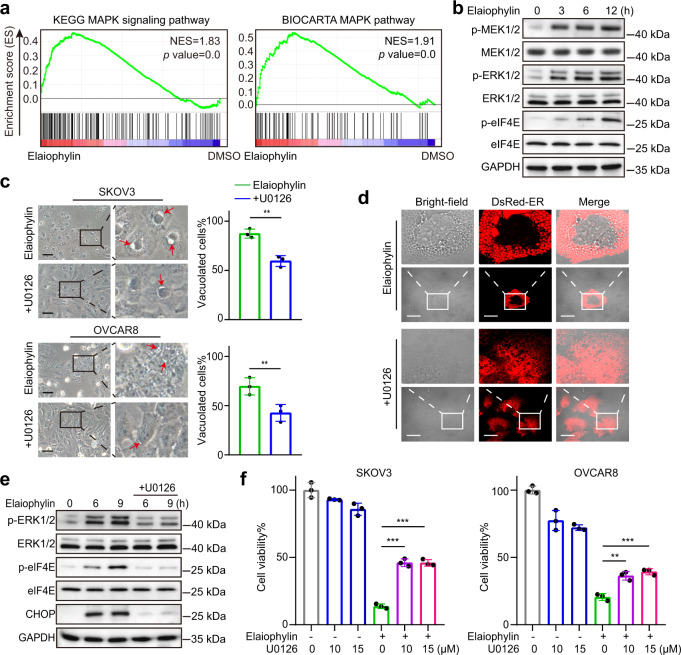
Paraptosis triggered by elaiophylin is mediated by MAPKs. **a** Gene set enrichment analysis of MAPK pathway in SKOV3 cells exposed to elaiophylin. **b** Assessment of indicated protein levels using western blotting in SKOV3 cells exposed to 0.5 µM elaiophylin. **c** Left panel: light microscopy images of SKOV3 and OVCAR8 cells exposed to 0.5 µM elaiophylin alone or in combination with U0126 (15 µM) for 9 h. Arrows indicate cytoplasmic vacuoles. Scale bar: 50 µm. Right panel: proportion of cells displaying vacuoles was scored by visually examining at least 100 cells. Data are mean ± SD of three independent experiments (Two-tailed unpaired Student’s *t* test, **p < 0.01). **d** Representative fluorescence and bright-field images of SKOV3 cells expressing DsRed-ER exposed to 0.5 µM elaiophylin alone or in combination with U0126 (15 µM) for 9 h. Scale bar: 20 µm. **e** Assessment of indicated protein levels using western blotting in SKOV3 cells exposed to 0.5 µM elaiophylin alone or in combination with U0126 (15 µM). **f** The viability of SKOV3 cells exposed to 0.5 µM elaiophylin alone or in combination with U0126 for 36 h. Data are mean ± SD of three independent experiments (Two-tailed unpaired Student’s *t*-test, **p < 0.01, ***p < 0.001)

MAPK activation has been reported to be another feature of paraptosis. GSEA indicated that the MAPK signaling pathway was significantly enriched in the elaiophylin-treated groups (Fig. 3a). The phosphorylation levels of ERK1/2 and MEK1/2 markedly increased in elaiophylin-treated SKOV3 cells (Fig. 3b). eIF4E, the translation initiator acting downstream of MAPKs,^24,25^ was also significantly phosphorylated (Fig. 3b), indicating that elaiophylin-induced MAPK activation promoted nascent protein synthesis. These data further confirm the paraptotic cell death induced by elaiophylin.

Next, U0126, a MEK1/2 inhibitor, was used to investigate whether MEK/ERK activation was responsible for elaiophylin-induced ER stress and paraptosis. We observed that U0126 distinctly diminished elaiophylin-induced vacuolization in SKOV3 and OVCAR8 cells (Fig. 3c). The elaiophylin-induced ER dilatation observed in SKOV3-DsRed-ER cells was also markedly eliminated by U0126 (Fig. 3d). Western blotting revealed that ERK1/2 and eIF4E were less phosphorylated and the ER stress indicator CHOP was less upregulated in the presence of U0126 in elaiophylin-treated SKOV3 cells (Fig. 3e). Additionally, U0126 partially attenuated elaiophylin-induced cell death (Fig. 3f). Furthermore, simultaneous knockdown of MEK1/2 (*MAP2K1*/*MAP2K2*, mitogen-activated protein kinase kinase 1/2) or ERK1/2 (*MAPK3*/*MAPK1*, mitogen-activated protein kinase 3/1) both occluded elaiophylin-induced vacuolization, ER stress, and cell viability loss (Supplementary Fig. S7). In summary, these results suggest that elaiophylin-induced paraptosis is mediated by MAPKs.

### CRISPR library screening identifies *PTPN11* as a critical gene for elaiophylin-induced paraptosis

**Fig. 4:**
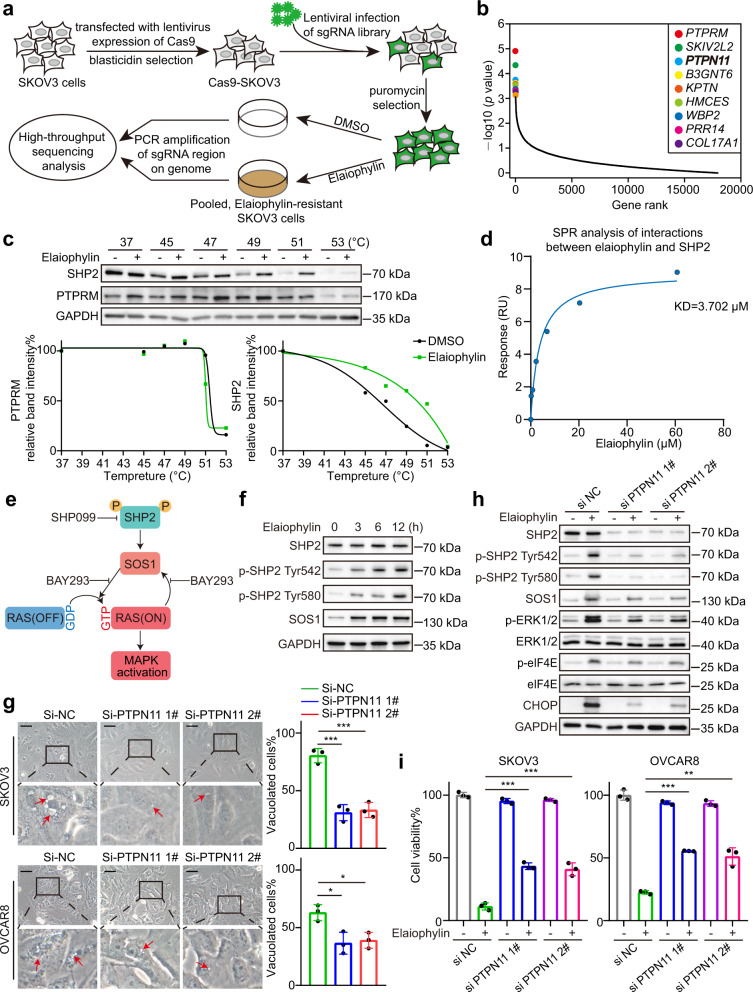
CRISPR library screening identifies *PTPN11* as a critical gene for elaiophylin-induced paraptosis. **a** A diagram for the genome-wide CRISPR/Cas9 knockout library screening. **b** Elaiophylin selection identified potential target genes. The top 10 positive selected genes were ranked by *p* value. **c** Top: SKOV3 cells were exposed for 24 h to DMSO or 0.5 µM elaiophylin and subject to CETSA. Bottom: the intensity of the SHP2 bands or PTPRM bands was quantified to exhibit the binding affinity of elaiophylin to SHP2 or PTPRM in SKOV3 cells. **d** SPR analysis showing direct interaction between elaiophylin and human recombinant SHP2 protein at the molecular level. **e** Pattern diagram of SHP2/SOS1/MAPK pathway. **f** Assessment of indicated protein levels using western blotting in SKOV3 cells exposed to 0.5 µM elaiophylin. **g** Left panel: representative light microscopy images of SKOV3 and OVCAR8 cells exposed to 0.5 µM elaiophylin for 9 h after *PTPN11* knockdown. Arrows indicate cytoplasmic vacuoles. Scale bar: 50 µm. Right panel: proportion of cells displaying vacuoles was scored by visually examining at least 100 cells. Data are mean ± SD of three independent experiments (Two-tailed unpaired Student’s *t* test, *p < 0.05, ***p < 0.001). **h** Assessment of indicated protein levels using western blotting in SKOV3 cells exposed to 0.5 µM elaiophylin for 9 h after *PTPN11* knockdown. **i** The viability of SKOV3 and OVCAR8 cells exposed to 0.5 µM elaiophylin for 36 h after *PTPN11* knockdown. Data are mean ± SD of three independent experiments (Two-tailed unpaired Student’s *t* test, **p < 0.01, ***p < 0.001)

To identify the critical genes involved in elaiophylin-induced paraptosis, we performed genome-wide CRISPR/Cas9 knockout library screening. SKOV3-Cas9 cells were transduced with the Human GeCKO v2 Library. After puromycin selection, cells expressing gRNAs were split into duplicates and treated with dimethyl sulfoxide (DMSO) or elaiophylin. After 10 days of treatment, the surviving cells were collected, gRNAs were amplified, and amplicons were sequenced (Fig. 4a). The top ten candidates were identified for positive selection (Fig. 4b). Positive selection identified candidate genes whose deletions led to elaiophylin resistance, indicating that these genes were potential targets of elaiophylin. Among these, *PTPRM* (protein tyrosine phosphatase receptor type M) and *PTPN11* (protein tyrosine phosphatase non-receptor type 11, encoding SHP2, Src homology 2 domain-containing tyrosine phosphatase 2) are related to MAPK signaling. To determine the direct target of elaiophylin, we performed a cellular thermal shift assay (CETSA) to evaluate the binding affinity of elaiophylin towards PTPRM or SHP2 protein. Elaiophylin treatment shifted the SHP2 melting curve to the right, rather than the PTPRM melting curve (Fig. 4c), implying direct binding between elaiophylin and SHP2 protein. Next, we employed surface plasmon resonance (SPR) to further confirm that elaiophylin bound to the recombinant SHP2 protein in a dose-dependent manner (Fig. 4d).

SHP2 activates the RAS/MAPK pathway by promoting RAS conversion from the inactive GDP-bound form to the active GTP-bound form via SOS1 (Son of sevenless homolog 1).^26,27^ In the meantime, SOS1 itself is activated by RAS, forming a positive feedback loop between SOS1 and RAS, which upregulates RAS/MAPK signaling (Fig. 4e). Next, we investigated whether elaiophylin activated SHP2. In elaiophylin-treated cells, the phosphorylation of Tyr542/580 of SHP2 was markedly increased, with increased expression of SOS1, indicating that elaiophylin may activate the MAPK pathway by SHP2 (Fig. 4f). To further validate whether SHP2 is necessary for elaiophylin-induced paraptosis, siRNAs against *PTPN11* were used. *PTPN11* knockdown distinctly attenuated elaiophylin-induced vacuolization in SKOV3 and OVCAR8 cells (Fig. 4g). *PTPN11* knockdown also prevented the elaiophylin-induced increase in p-ERK1/2, p-eIF4E, CHOP (Fig. 4h), and viability loss (Fig. 4i).

To further verify that SHP2/SOS1/MAPK pathway activation was required for elaiophylin-induced paraptosis, we used SHP099 (SHP2 inhibitor) and BAY293 (SOS1/RAS interaction inhibitor). As expected, SHP099 and BAY293 effectively decreased vacuolization and cell death in elaiophylin-treated cells (Supplementary Fig. S8a–d). Moreover, SHP099 and BAY293 prevented the elaiophylin-induced increase in p-ERK1/2, p-eIF4E, and CHOP (Supplementary Fig. S8e, f). In addition, the salvage effects of paraptosis-related inhibitors CHX, SHP099, and BAY293 were more prominent than those of the apoptosis inhibitor Z-VAD-FMK (Supplementary Fig. S9a, b). Meanwhile, *PTPN11* knockdown, SHP099, and BAY293 partially eliminated the cleavage of caspase3 and PARP (Supplementary Fig. S9c, d). Collectively, our results suggest that SHP2 is a critical target for elaiophylin-induced paraptosis.

### Elaiophylin-induced paraptosis blocks autophagy by impairing lysosomal function

**Fig. 5:**
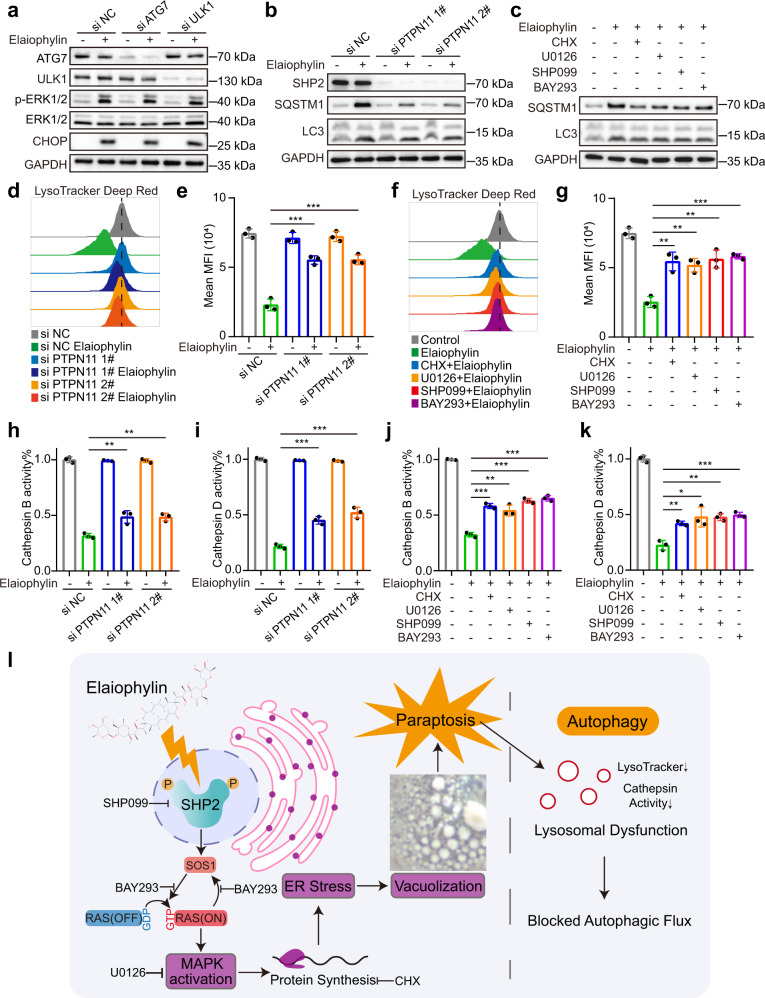
Elaiophylin-induced paraptosis partially contributes to autophagy inhibition by impairing lysosomal function. **a** Assessment of indicated protein levels using western blotting in SKOV3 cells exposed to 0.5 µM elaiophylin for 9 h after *ATG7* or *ULK1* knockdown. **b** Assessment of indicated protein levels using western blotting in SKOV3 cells exposed to 0.5 µM elaiophylin for 24 h after *PTPN11* knockdown. **c** Assessment of indicated protein levels using western blotting in SKOV3 cells exposed to 0.5 µM elaiophylin for 24 h alone, or in combination with CHX (5 µM pretreatment for 4 h), U0126 (15 µM), SHP099 (30 µM), BAY293 (5 µM), respectively. **d** FACS analysis of LysoTracker Deep Red in SKOV3 cells exposed to 0.5 µM elaiophylin for 24 h after *PTPN11* knockdown. **e** Mean fluorescence intensities in (**d**) were quantified. Data are mean ± SD of three independent experiments (Two-tailed unpaired Student’s *t* test, ***p < 0.001). **f** FACS analysis of LysoTracker Deep Red in SKOV3 cells exposed to 0.5 µM elaiophylin for 24 h alone, or in combination with CHX (5 µM pretreatment for 4 h), U0126 (15 µM), SHP099 (30 µM), BAY293 (5 µM), respectively. **g** Mean fluorescence intensities in (**f**) were quantified. Data are mean ± SD of three independent experiments (Two-tailed unpaired Student’s *t*-test, **p < 0.01, ***p < 0.001). **h**, **i** SKOV3 cells were exposed to 0.5 µM elaiophylin for 36 h after *PTPN11* knockdown, and subject to enzymatic activity determination of CTSB (**h**) and CTSD (**i**). Data are mean ± SD of three independent experiments (Two-tailed unpaired Student’s *t* test, **p < 0.01, ***p < 0.001). **j**, **k** SKOV3 cells were exposed to 0.5 µM elaiophylin alone for 36 h, or in combination with CHX (5 µM pretreatment for 4 h), U0126 (15 µM), SHP099 (30 µM), BAY293 (5 µM), respectively, and subject to enzymatic activity determination of CTSB (**j**) and CTSD (**k**). Data are mean ± SD of three independent experiments (Two-tailed unpaired Student’s *t* test, *p < 0.05, **p < 0.01, ***p < 0.001). **l** A schematic diagram of paraptosis and consequent autophagy inhibition induced by elaiophylin. Chemical structure depiction of elaiophylin was obtained from NCBI, PubChem Compound Summary for CID 6444206, Elaiophylin. Retrieved 2 January, 2022 from https://pubchem.ncbi.nlm.nih.gov/compound/Elaiophylin

As elaiophylin is an autophagy inhibitor, we sought to explore the relationship between paraptosis and autophagy. First, *ATG7* and *ULK1*, two critical autophagy genes, were knocked down using siRNAs to test the impact of autophagy regulation on elaiophylin-induced paraptosis. *ATG7* or *ULK1* knockdown did not prevent ERK1/2 phosphorylation and CHOP increase in response to elaiophylin (Fig. 5a). As mentioned above, elaiophylin-induced ER stress occurred earlier than autophagy inhibition in the temporal gradient assay (Fig. 2c). Therefore, we hypothesized that blocked autophagic flux was the outcome of paraptosis. To test this hypothesis, we regulated paraptosis using siRNAs against *PTPN11* and the inhibitors used above to examine their impacts on autophagy. As expected, *PTPN11* knockdown decreased SQSTM1 accumulation following elaiophylin treatment (Fig. 5b). Similarly, the addition of CHX, U0126, SHP099, or BAY293 partially eliminated SQSTM1 accumulation in elaiophylin-treated SKOV3 cells (Fig. 5c), implying that autophagy inhibition was partially restored by downregulated paraptosis.

Previously, we proved that elaiophylin inhibited autophagy by disrupting lysosomal function.^23^ We then tested whether paraptosis was responsible for elaiophylin-induced lysosomal dysfunction. First, *PTPN11* knockdown or the addition of CHX, U0126, SHP099, and BAY293 rescued the decrease in LysoTracker Deep Red fluorescence in SKOV3 cells exposed to elaiophylin (Fig. 5d–g), suggesting that the volume loss of the acidic compartment was restored. On the other hand, lysosomal dysfunction manifested as reduced enzymatic activity of cathepsin B (CTSB) and cathepsin D (CTSD). Consistently, *PTPN11* knockdown or addition of CHX, U0126, SHP099, and BAY293 partially rescued the impaired activity of CTSB and CTSD (Fig. 5h–k). Collectively, these data suggest that paraptosis at least partially contributes to elaiophylin-induced autophagy inhibition by impairing lysosomal function (Fig. 5l).

### Elaiophylin induces paraptosis and consequent autophagy inhibition in vivo

**Fig. 6:**
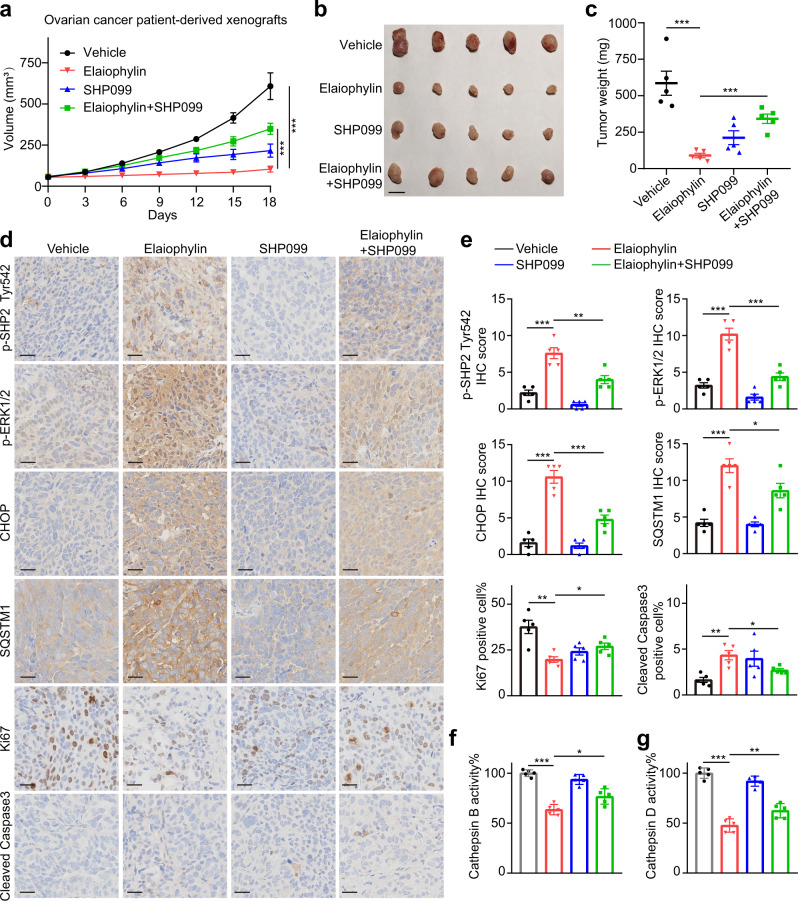
SHP2 inhibition attenuates the effects of elaiophylin in PDX models. **a** PDX models were established on female NOG mice using the tumor tissues from a high-grade serous ovarian cancer patient, and were treated with vehicle, elaiophylin (2 mg/kg/d), SHP099 (30 mg/kg/d), or the combination of elaiophylin (2 mg/kg/d) and SHP099 (30 mg/kg/d) (*n* = 5 in each group). From the beginning of treatment, tumor volumes were measured to plot tumor growth curves. Data are mean ± SEM (Two-tailed unpaired Student’s *t* test, ***p < 0.001). **b** Photograph of resected tumor tissues from PDX models. Scale bar: 1 cm. **c** Quantification of tumor weight in (**b**). Data are mean ± SEM (Two-tailed unpaired Student’s *t* test, ***p < 0.001). **d** Representative images of immunohistochemical staining with indicated antibodies in tumor specimens from (**b**). Scale bar: 25 µm. **e** Quantification of immunohistochemical scores in (**d**). Five sections were assessed per group and the mean of four randomly selected viewing fields were evaluated for every section. Data are mean ± SEM (Two-tailed unpaired Student’s *t*-test, *p < 0.05, **p < 0.01, ***p < 0.001). **f**, **g** Enzymatic activity of CTSB (**f**) and CTSD (**g**) in tumor tissues from (**b**). The results are presented as percentages of the vehicle group. Data are mean ± SEM (Two-tailed unpaired Student’s *t* test, *n* = 5, *p < 0.05, **p < 0.01, ***p < 0.001)

Using an ovarian cancer patient-derived xenograft (PDX) model, we validated the significant antitumor effects of elaiophylin, which could be counteracted by SHP099 (Fig. 6a–c). Besides, the IHC staining of PDX models phenocopied in vitro studies. Specifically, elaiophylin increased the expression of p-SHP2 Tyr 542, p-ERK1/2, CHOP, SQSTM1, cleaved caspase 3, and decreased Ki67 expression, which were reversed by combination with SHP099 (Fig. 6d, e). In addition, cathepsin activity assays conducted on tumor tissues revealed a significant decrease in CTSB and CTSD enzymatic activity in the elaiophylin treatment group, which was also rescued by SHP099 (Fig. 6f, g), indicating that SHP2 inhibition partially abolished elaiophylin-induced paraptosis, lysosomal dysfunction, autophagy inhibition, and apoptosis in vivo.

Next, we assessed the antitumor effect of elaiophylin in an immunocompetent ID8 murine ovarian cancer model. Similarly, elaiophylin activated the SHP2/SOS1/MAPK pathway and induced ER stress and cytoplasmic vacuoles in ID8 cells (Supplementary Fig. S10a–c). Gradient doses of elaiophylin (2, 4, and 6 mg/kg) showed powerful antineoplastic capabilities, as evidenced by decreased tumor volumes and weights (Supplementary Fig. S10d). In addition, tumor specimens from the elaiophylin treatment group demonstrated dose-dependent vacuolization and ER dilation in tumor cells (Supplementary Fig. S10e, f). IHC analysis of ID8 tumors demonstrated that p-SHP2 Tyr 542, p-ERK1/2, CHOP, SQSTM1, and cleaved Caspase3 were increased in elaiophylin-treated tumors, but p-MLKL, 4-HNE, GPX4, and HMOX1 staining presented no upregulation, indicating decreased Ki67 expression in elaiophylin treatment group was associated with the induction of paraptosis, apoptosis and autophagy inhibition rather than ferroptosis or necroptosis (Supplementary Fig. S10g, h). Furthermore, cathepsin activity assays conducted on tumor tissues revealed a dose-dependent decrease in CTSB and CTSD enzymatic activity (Supplementary Fig. S10i, j). Moreover, there was no weight loss or evident organ toxicities in mice treated with 2, 4, and 6 mg/kg elaiophylin (Supplementary Fig. S11), indicating that these doses were tolerable in *vivo*. These data confirm that elaiophylin is also effective in immunocompetent mouse model.

### Elaiophylin exploits MAPK activation as a vulnerability in drug-resistant ovarian cancer

**Fig. 7:**
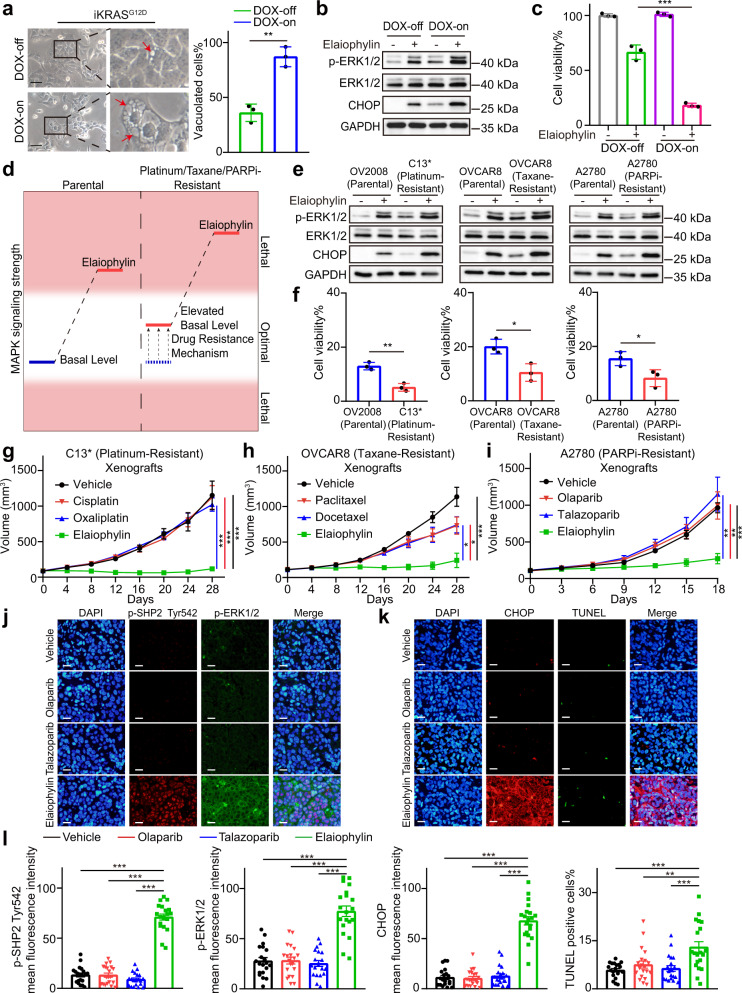
Elaiophylin exploits MAPK activation as a vulnerability in drug-resistant ovarian cancer. **a** Left panel: representative light microscopy images of doxycycline (DOX)-inducible iKRAS cells cultured in the absence or presence of DOX (2 μg/mL) for 48 h and exposed to 0.75 µM elaiophylin for 6 h. Arrows indicate cytoplasmic vacuoles. Scale bar: 50 µm. Right panel: proportion of cells displaying vacuoles was scored by visually examining at least 100 cells. Data are mean ± SD of three independent experiments (Two-tailed unpaired Student’s *t* test, **p < 0.01). **b** Assessment of indicated protein levels using western blotting in iKRAS cells cultured in the absence or presence of DOX (2 μg/mL) for 48 h and then exposed to 0.75 µM elaiophylin for 6 h. **c** The viability of iKRAS cells cultured in the absence or presence of DOX (2 μg/mL) for 48 h and then exposed to 0.75 µM elaiophylin for 24 h. Data represent three independent experiments and are shown as mean ± SD (Two-tailed unpaired Student’s *t* test, ***p < 0.001). **d** A schematic diagram of elaiophylin-induced MAPK activation in parental and resistant cells. **e** Assessment of indicated protein levels using western blotting in indicated parental and resistant cells exposed to 0.5 µM elaiophylin for 6 h. **f** The viability of indicated parental and resistant cells exposed to 0.5 µM elaiophylin for 36 h. The results are presented as percentages of the control. Data are mean ± SD of three independent experiments (Two-tailed unpaired Student’s *t* test, *p < 0.05, **p < 0.01). **g** Female immune-deficient BALB/c nu/nu (nude) mice were inoculated subcutaneously with C13* cells and were treated with vehicle, cisplatin (10 mg/kg/2d), oxaliplatin (10 mg/kg/2d), or elaiophylin (2 mg/kg/d) (*n* = 5 in each group). From the beginning of treatment, tumor volumes were measured to plot tumor growth curves. Data are mean ± SEM (Two-tailed unpaired Student’s *t* test, ***p < 0.001). **h** Female immune-deficient BALB/c nu/nu (nude) mice were inoculated subcutaneously with OVCAR8 (taxane-resistant) cells and were treated with vehicle, paclitaxel (30 mg/kg/2d), docetaxel (30 mg/kg/2d), or elaiophylin (2 mg/kg/d) (*n* = 5 in each group). From the beginning of treatment, tumor volumes were measured to plot tumor growth curves. Data are mean ± SEM (Two-tailed unpaired Student’s *t* test, *p < 0.05, ***p < 0.001). **i** Female immune-deficient BALB/c nu/nu (nude) mice were inoculated subcutaneously with A2780 (PARPi-resistant) cells and were treated with vehicle, olaparib (50 mg/kg/d), talazoparib (0.33 mg/kg/d), or elaiophylin (2 mg/kg/d) (*n* = 5 in each group). From the beginning of treatment, tumor volumes were measured to plot tumor growth curves. Data are mean ± SEM (Two-tailed unpaired Student’s *t* test, **p < 0.01, ***p < 0.001). **j** Sections of tumors from (**i**) were stained using p-SHP2 Tyr542 antibody (red), p-ERK1/2 antibody (green), and DAPI (blue). Representative sections are shown. Scale bar: 20 µm. **k** Sections of tumors from (**i**) were stained using CHOP antibody (red), DAPI (blue), and using TUNEL assay (green). Representative sections are shown. Scale bar: 20 µm. **l** Quantification of the mean fluorescence intensity and TUNEL-positive proportion in (**j**) and (**k**). Four randomly selected viewing fields were individually evaluated per section and five sections were assessed per group. Data are mean ± SEM (Two-tailed unpaired Student’s *t* test, **p < 0.01, ***p < 0.001)

Since elaiophylin triggers paraptotic cell death by activating the MAPK pathway, we set out to explore the impact of MAPK basal levels on elaiophylin-induced paraptosis by using iKRAS^G12D^ cells, a KRAS-inducible cell line where RAS/MAPK activation was inducible with doxycycline (DOX).^28^ Interestingly, elaiophylin-treated DOX-on iKRAS cells exhibited more extensive cytoplasmic vacuolization (Fig. 7a), significantly higher p-ERK1/2 and CHOP expression (Fig. 7b), and more viability loss (Fig. 7c) than their DOX-off counterparts. In addition, normal cells were less affected by elaiophylin than cancer cells (Supplementary Fig. S12). Furthermore, a significant positive correlation was observed between the basal levels of MAPK and elaiophylin-induced cell death in the eight cancer cell lines used in this study (Supplementary Fig. S13), suggesting that elaiophylin-induced paraptosis was highly dependent on basal levels of MAPK signaling activity.

Given that MAPKs are often aberrantly activated during drug resistance acquisition in multiple cancers,^17,18,29,30^ we wondered whether elaiophylin would be more effective against drug-resistant ovarian cancer. To validate this hypothesis (Fig. 7d), we used the cisplatin-sensitive cell line OV2008 and its cisplatin-resistant derivative C13* cells (Supplementary Fig. S14a). In addition, taxane-resistant OVCAR8 clones (Supplementary Fig. S14b) and PARPi-resistant A2780 clones were generated (Supplementary Fig. S14c) by culturing the cells in the presence of paclitaxel or olaparib for three months. These drug-resistant cell lines exhibited higher basal levels of MAPK than their parental cells. Elaiophylin treatment displayed increased cytotoxicity and autophagy inhibition in drug-resistant derivatives than in parental cells (Fig. 7e, f and Supplementary Fig. S15), indicating that as ovarian cancer cells became resistant to platinum, taxane, or PARPi, they simultaneously became hypersensitized to elaiophylin.

Next, the antitumor effects of elaiophylin were investigated in vivo using three drug-resistant models: platinum-resistant C13* xenografts, taxane-resistant OVCAR8 xenografts, and PARPi-resistant A2780 xenografts. In these models, chemotherapy or PARPi failed to suppress tumor growth, whereas 2 mg/kg elaiophylin significantly controlled tumor growth (Fig. 7g–i and Supplementary Fig. S16a–i). H&E staining of tumor sections showed typical features of vacuolization in the elaiophylin-treated groups (Supplementary Fig. S16j). Immunofluorescence analysis of tumor sections demonstrated that p-SHP2 Try542, p-ERK, and CHOP expression were strikingly increased, accompanied by moderately increased TUNEL staining in elaiophylin-treated xenografts compared with the other groups (Fig. 7j–l and Supplementary Fig. S17), suggesting that elaiophylin fully activated the SHP2/MAPK pathway and induced ER stress in chemotherapy- and PARPi-resistant tumors. IHC analysis of markers of cell death and proliferation in drug-resistant tumors phenocopied those in ID8 tumors after elaiophylin treatment (Supplementary Figs. S18, 19, 20). Taken together, we conclude that elaiophylin-induced paraptosis overcomes chemotherapy resistance and PARPi resistance by exploiting MAPK activation as a vulnerability factor in ovarian cancer.

## Discussion

Inducing nonapoptotic cell death in cancer cells is regarding as a promising antitumor strategy.^31–33^ In this study, we revealed that elaiophylin could induce excessive ER stress, ER-derived vacuolization, and consequent paraptosis in ovarian cancer cells. An unbiased genome-wide CRISPR/Cas9 knockout library screening, CETSA, and SPR identified the direct interaction between elaiophylin and SHP2, and overactivation of the SHP2/SOS1/MAPK pathway as the underlying mechanism. Most importantly, increased MAPK levels in drug-resistant ovarian cancers, which offered them survival advantage against chemotherapy and PARPi, became an Achilles’s heel for elaiophyin to unleash cytotoxicity by inducing MAPK overactivation (Supplementary Fig. S21).

Previously, we screened and identified elaiophylin as a late-stage autophagy inhibitor,^23^ which was later confirmed in multiple tumor models.^34,35^ Initiated by massive cytoplasmic vacuoles not overlaying autophagosomes, we unveiled the capability of elaiophylin to provoke paraptosis. Although apoptosis accounted for a part of elaiophylin-induced cell death, the weak rescue of the apoptosis inhibitor suggested that apoptosis was not the principal mechanism of action. Consistent with our findings, autophagosome accumulation is frequently observed in cancer cells treated with several other paraptosis inducers,^36,37^ whereas the correlation between these two vital biological processes has not been fully elucidated. Our previous article provided a thorough justification to certify that elaiophylin blocks autophagic flux at the late stage by attenuating lysosomal function.^23^ Accordingly herein, we found paraptosis, at least partially, responsible for elaiophylin-induced lysosomal dysfunction and resulting autophagy inhibition, revealing the detailed relationship between paraptosis and autophagy.

The induction of paraptotic cell death is emerging as a potential direction for antitumor therapy.^21^ To date, numerous natural compounds have been identified as paraptosis inducers, such as curcumin and celastrol.^36,38^ In addition, some synthetic compounds could also induce paraptosis, such as benzo[a]quinolizidine analogs and nanomedicines made from Cu^2+^-responsive HQ-releasing micelles (Cu(HQ)_2_).^31,37^ However, the mechanisms underlying paraptosis are insufficiently understood, although some features including activation of MAPKs, de novo protein synthesis, and ER stress have been described.^20^ Here, we systematically explored the role of MAPKs in elaiophylin-induced paraptosis. First, MEK inhibitors, or siRNAs targeting MEK1/2 or ERK1/2 effectively abrogated elaiophylin-induced ER stress, ER-derived vacuoles, and paraptotic cell death. Second, using genome-wide CRISPR/Cas9 knockout library screening, SHP2 (encoded by *PTPN11*), an RAS/MAPK pathway activator, was identified as an essential target of elaiophylin-induced paraptosis. Inhibition of the SHP2/SOS1/MAPK pathway by siRNAs directed against *PTPN11* or pharmacological inhibitors distinctly attenuates paraptosis. Third, activation of the RAS/MAPK pathway with DOX in iKRAS^G12D^ cells sensitizes the cells to elaiophylin-triggered paraptosis. The different reactivities of paired cells with different basal MAPK levels profoundly reflected the decisive nature of MAPKs in paraptotic cell death.

Over the past two decades, numerous pathways or targets have been regarded as loss-of-control mechanisms of cancer, such as DNA replication stress,^39^ reactive oxygen species,^40^ driver mutations,^41^ and metabolic reprogramming.^42^ Traditional therapeutics aim to curb or even reverse these hallmarks, that is, “shutting off the engine”.^43^ In contrast, a sophisticated concept is to “fuel the engine” to an unsustainable extent until cancer cells collapse. Consistent with this concept, in the present study, we demonstrated that elaiophylin directly acted on SHP2 and hyperactivated MAPK signaling, thus triggering paraptosis. MAPK activation liberated ovarian cancer cells from cytotoxicity of platinum, taxane and PARP inhibitors, but contemporaneously became a vulnerability to elaiophylin-induced paraptosis. Thus, a thorough understanding of cellular homeostasis management reveals the potential of such novel approaches in overcoming resistance by turning the advantages of malignancies into fatal flaws.

In summary, we demonstrated that elaiophylin triggers paraptosis by hyperactivating the SHP2/SOS1/MAPK pathway, inducing excessive ER stress and ER-derived vacuolization. Exploiting the elevated basal MAPK levels in drug-resistant cells, elaiophylin overcame platinum, taxane, and PARPi resistance in ovarian cancer. The notable effects and safety of elaiophylin in mouse models observed in this study support the further investigation of elaiophylin in clinical trials and strengthen the idea that paraptosis inducers might represent a novel treatment approach, especially in drug-resistant tumors with enhanced MAPK activities.

## Materials and methods

### Ethics declarations

The acquisition of clinical specimens was approved by the Tongji Hospital Ethics Committee, and the consents were obtained from the participants before the study. Animal studies were approved by the Tongji Hospital Laboratory Animal Welfare and Ethics Committee. All animal experiments were carried out according to the principles of the Declaration of Helsinki.

### Compounds and antibodies

Elaiophylin was obtained from NCPGC (North China Pharmaceutical Group Corporation) New Drug Research & Development Center (Hebei, China). Cycloheximide (HY-12320), SHP099 (HY-100388), BAY-293 (HY-114398), cisplatin (HY-17394), oxaliplatin (HY-17371), paclitaxel (HY-B0015), and docetaxel (HY-B0011) were purchased from MCE (New Jersey, USA). CQ (C6628) was purchased from Sigma-Aldrich (St. Louis, MO). 3-MA (S2767), U0126 (S1102), olaparib (S1060), talazoparib (S7048), and DOX (S5159) were purchased from Selleck (Texas, USA). Primary antibodies against CHOP (2895), p-ERK1/2 (4696), p-MEK1/2 (Ser217/221, 9154), Erk1/2 (4695), MEK1/2 (8727), p-eIF4E (Ser209, 9741), SHP2 (3397), p-SHP2 (Tyr580, 3703), SOS1 (5890), RIPK1 (3493), p-RIPK1 (Ser166, 65746), Caspase3 (9662), Cleaved Caspase3 (9664), EpCAM (2929), and ULK1 (8054) were purchased from CST (Cell Signaling Technology, MA, USA). Primary antibodies against p-IRE1 (S724, ab48187), p-SHP2 (Y542, ab62322), Ki67 (ab16667), MLKL (ab184718), MLKL (S345, ab196436, mouse reactivity), MLKL (S358, ab187091, human reactivity), GSDMD (ab210070), GSDME (ab215191), and ATG7 (ab133528) were purchased from Abcam (Cambridge, UK). Primary antibodies against ATF4 (10835-1-AP), GRP78 (11587-1-AP), Alix (12422-1-AP), SQSTM1 (18420-1-AP), LC3 (14600-1-AP), eIF4E (11149-1-AP), GPX4 (67763-1-Ig), Calnexin (10427-2-AP), PAX8 (10336-1-AP), and GAPDH (10494-1-AP) were purchased from Proteintech (Hubei, China). Primary antibody against PTPRM (YN2112) was purchased from Immunoway (Jiangsu, China).

### Cell lines and cell culture

SKOV-3, SW626, and UWB1.289 cells were obtained from the American Type Culture Collection. OVCAR8 cells were obtained from the MDACC-characterized cell line core. A2780 cells were purchased from European Collection of Authenticated Cell Cultures. The OV2008 and C13* cell lines were gifts from Professor Benjamin K. Tsang of the Ottawa Health Research Institute (Ottawa, Canada).^44^ iKRAS cells, primary murine pancreatic cells established from p48 Cre_tetO_LKras^G12D^ ROSA_rtTAL^+^p53L^+^ mice, were gifted by Professor Anirban Maitra (MDACC, Houston, TX, USA). All cell lines were tested monthly for *Mycoplasma* using PCR and were not passaged more than 30 times.

SKOV3 cells were cultured in McCoy’s 5A medium supplemented with 10% FBS. SW626 cells were cultured in L-15 medium supplemented with 10% fetal bovine serum (FBS). UWB1.289 cells were cultured in a medium containing RPMI-1640 and MEGM (Lonza, CC-3150) mixed at a 1:1 ratio with 3% FBS. SW626 cells were cultured in L-15 medium supplemented with 10% FBS. OVCAR8, A2780, OV2008, C13*, and iKRAS cells were cultured in RPMI-1640 medium supplemented with 10% FBS. All cell lines except SW626 were cultured at 37 °C in a 5% (v/v) CO_2_ atmosphere. SW626 cells were cultured at 37 °C in a free gas exchange with atmospheric air.

### Generation of drug-resistant clones

A2780 cells were exposed to a gradual increase in olaparib concentrations until the cells grew in the presence of 12 μM of the drug (3 months from initial exposure). Cells were then cultured without olaparib for one month before use in the experiments.

OVCAR8 cells were cultured with a gradual increase in paclitaxel concentrations until the cells grew in the presence of 10 nM of the drug (3 months from initial exposure). Cells were cultured without paclitaxel for one month before use in the experiments.

C13* cells are cisplatin-resistant derivatives of the cisplatin-sensitive cell line OV2008 cultivated by monthly in vitro selection with cisplatin.^44^

### Microscopy

For cytoplasmic vacuolization, images were taken using an Olympus DP80 fluorescence microscope. GFP-LC3B, GFP-Mito, and DsRed-ER fluorescence images were captured using a laser scanning confocal microscope (Olympus FV1000).

### Transmission electron microscopy assay

After the treatments, cells were harvested and fixed with 2.5% glutaraldehyde overnight at 4 °C. Subsequently, the samples were post-fixed in 1% osmium tetroxide for 60 min, then embedded and sectioned. The sections were double-stained with uranyl acetate and lead citrate and observed using a transmission electron microscope (FEI company).

### Cell viability assay

Cells were seeded into 96-well plates and cultured overnight. After each treatment, cell viability was determined by Cell Counting Kit-8 (CCK8, Dojindo Laboratories) assay according to the manufacturer’s instruction.

### Gene expression profiling and GSEA

Gene expression microarray data of SKOV3 cells exposed to elaiophylin was taken from our previous study, and GSEA was performed as previously described.^23^

### Western blotting

Cells were lysed in RIPA buffer (Servicebio, G2002-100) containing protease inhibitors (Topscience, C0001) and phosphatase inhibitors (Topscience, C0004). After thorough mixing and incubation at 4 °C for 30 min, extracts were sonicated for 30 s and clarified by centrifugation at 12000 × *g* and 4 °C for 25 min. The protein content was determined, loaded onto 10% SDS-PAGE gels, and transferred onto 0.45 μm PVDF membranes. After blocking with 5% BSA, the membranes were probed with primary antibodies at 4 °C overnight, followed by incubation with a horseradish peroxidase-labeled secondary antibody (Antgene, ANT019, ANT020). Signals were visualized using a WesternBright^TM^ ECL kit (Advansta, 190113-13) and enhanced chemiluminescence (Bio-Rad).

### Real-time quantitative PCR (qPCR)

Total RNA (1 μg) isolated from the indicated samples was reverse-transcribed using the PrimeScript™ RT reagent Kit (Takara). The cDNA was subjected to qPCR using the iTAQ™ Universal SYBR^®^ Green Supermix (Bio-Rad). Target gene expression was normalized to that of GAPDH and calculated using the delta-delta-Ct (ddCt) method. The primer sets used were as follows: ATF3 (forward, CCTCTGCGCTGGAATCAGTC; reverse, TTCTTTCTCGTCGCCTCTTTTT), CHOP (forward, GGAAACAGAGTGGTCATTCCC; reverse, CTGCTTGAGCCGTTCATTCTC), HERP (forward, ATGGAGTCCGAGACCGAAC; reverse, TTGGTGATCCAACAACAGCTT), TRIB3 (forward, GCCTTTTTCACTCGGACCCAT; reverse, CAGCGAAGACAAAGCGACAC), GAPDH (forward, GGAGCGAGATCCCTCCAAAAT; reverse, GGCTGTTGTCATACTTCTCATGG).

### siRNA and plasmid

For siRNA transfection, cells were seeded into six-well plates at 30% confluency and transfected with siRNA using Lipofectamine 3000 (Invitrogen, L3000075). siRNAs targeting *PTPN11* (1# CAGACAGAAGCACAGUACCGAUUUA, 2# GAAAGGGCACGAAUAUACAAAUAUU) and scramble control siRNA (12935200) were designed and synthesized by Invitrogen. siRNAs targeting *ULK1* (GCCTGTTCTACGAGAAGAA), *ATG7* (GAACGAGTATCGGCTGGAT), *MAPK1* (GAACATCATTGGAATCAAT), *MAPK3* (CCTCCAACCTGCTCATCAA), *MAP2K1* (GAGGTTCTCTGGATCAAGT), and *MAP2K2* (TGGACTATATTGTGAACGA) were designed and synthesized by RiboBio.

For plasmid transfection, cells were seeded into six-well plates at 30% confluency and transfected with plasmids using HP (Roche, 47244400). GFP-LC3B plasmid was obtained from Beyotime (D2815). The GFP-Mito plasmid was obtained from Origene (RC100092) while the DsRed-ER plasmid was obtained from Addgene (# 55836).

### Genome-wide CRISPR/Cas9 knockout library screening

The human GeCKO v2 CRISPR Library (two Plasmid System-lentiGuide-Puro) was used in this study. The library was purchased from Addgene (1000000049). A stable Cas9-expressing SKOV3 cell line (SKOV3-Cas9) was established by transfection with lenti-Cas9 viruses, followed by blasticidin selection. SKOV3-Cas9 cells were transduced with the Human GeCKO v2 Library containing 10^5^ gRNAs targeting 19,050 human genes, with six gRNAs per gene at an MOI of ~0.3. After puromycin selection, cells expressing gRNAs were split into duplicates and treated with DMSO or elaiophylin. After 10 days of treatment, the remaining cells were collected. Genomic RNAs were extracted and amplified, and the amplicons were sequenced using next-generation sequencing (Illumina). Data analysis was performed using MAGeCK software.

### Cellular thermal shift assay (CETSA)

SKOV3 cells were exposed to DMSO or elaiophylin for 24 h, collected, washed with PBS containing protease inhibitors, aliquoted into PCR tubes, and heated in a thermal cycler (Bio-Rad, T100) at the indicated temperature for 3 min to denature proteins. The cells were then resuspended in NP40 buffer, subjected to three freeze-thaw cycles with liquid nitrogen, and centrifuged at 20,000 × *g* for 20 min at 4 °C. The supernatant was boiled in the loading buffer for western blotting.

### Surface plasmon resonance (SPR) assay

The SPR binding assay was performed using a Biacore-8K at 25 °C and a CM5 sensor. Human SHP2 protein (Abcam, ab227396) was immobilized onto a CM5 sensor at a density of 2000 RU at pH 4.5. The analyte elaiophylin was directly solubilized in running buffer (20 mM HEPES pH 7.4, 150 mM NaCl, and 0.05% surfactant P20) with a maximal concentration of 60 µM, and another five different concentration points were prepared in a 3-fold dilution series using the running buffer. Biacore insight evaluation software (version 3.0) was used to determine the dissociation affinity constant (KD) by fitting the response at each concentration at equilibrium using a steady-state affinity mode.

### LysoTracker Deep Red staining

Cells were collected and incubated with LysoTracker Deep Red (Invitrogen, L12492) at 37 °C for 30 min, protected from light. The stained cell suspensions were processed for flow cytometry using CytoFlex (Beckman Coulter, A00-1-1102). The data were analyzed using FlowJo v10 software.

### Cathepsin activity assay

Cathepsin activity was measured using the CTSB Activity Assay Kit (Abcam, ab65300) and the CTSD Activity Assay Kit (Abcam, ab65302). Cells and tissues were harvested and resuspended in chilled lysis buffer. Lysates were incubated with the substrate buffer solution at 37 °C for 1 h, while being protected from light. The output was measured using a fluorescence microplate reader. Enzyme activity was determined by comparing the results of the treated sample with those of the control sample.

### Immunohistochemistry

Tumor sections were subjected to deparaffinization and antigen retrieval before goat serum blocking and then incubated overnight at 4 °C with primary antibodies. After incubation with the HRP-conjugated secondary antibody, DAB staining was performed to detect the target. Immunostaining intensity was scored by estimating the staining intensity (0 = no staining, 1 = weak staining, 2 = moderate staining, 3 = strong staining) and the percentage of positive cells (0 = no staining, 1 = less than 10% staining, 2 = 10–25% staining, 3 = 26–50% staining, 4 = 51–75% staining, 5 = more than 75% staining). For every section, the mean of the five readings was calculated. The immunohistochemistry scores were assessed separately by two experts.

For hematoxylin and eosin staining, the deparaffinized sections were incubated in hematoxylin solution for 3 min, followed by 15 s of incubation with eosin.

### Immunofluorescence and TUNEL assay

For dual immunofluorescence staining, tumor sections were deparaffinized, rehydrated, and the antigen was recovered, permeabilized, and blocked with 5% BSA at room temperature for 1 h. The sections were incubated with primary antibodies at 4 °C overnight, followed by Alexa-Fluor conjugated secondary antibodies (Antgene, ANT023, ANT030) at room temperature for 1 h. Mounting was performed using a fluormount containing DAPI (Antgene, ANT063) for further image acquisition (Olympus BX53).

For TUNEL and immunofluorescence co-staining, tumor sections were deparaffinized, rehydrated, and the antigen was recovered, permeabilized, and blocked with 5% BSA for 1 h at room temperature. The sections were stained using a TUNEL assay kit (Vazyme, A112) according to the manufacturer’s instructions and then incubated with primary antibody at 4 °C overnight, followed by incubation with Alexa-Fluor conjugated secondary antibody (Antgene, ANT029) at room temperature for 1 h. Mounting was performed using a fluormount containing DAPI (Antgene, ANT063) for further image acquisition (Olympus BX53).

Quantification of mean fluorescence intensity and TUNEL-positive proportion was performed using ImageJ software. Five sections were assessed in each group, and four randomly selected viewing fields were evaluated individually in each section.

### Primary cancer cell & HUMSC

Primary ovarian cancer cells were isolated from the tumor tissues of a high-grade serous ovarian cancer patient. Tumor tissues were dissected into cubes and dissociated by Tumor Dissociation Kit (Miltenyi, 130-095-929).

For HUMSC, fresh umbilical cord tissues were washed in HBSS buffer, dissected into cubes, and transferred into culture dishes to allow the migration of HUMSC. After the third passage, the cells were identified and used for experiments.

### Animal studies

Female BALB/c nude mice (4–5 weeks old), female C57BL-6J mice (4–5 weeks old), and female NOG mice (6 weeks old) were purchased from Beijing Vital River Laboratory Animal Technology Co., Ltd., and housed under SPF (specific pathogen-free) conditions.

For the models established from cell line, cells (5 × 10^6^) were resuspended in a mixture of PBS and Matrigel (354234; Corning) and subcutaneously injected into mice. For PDX model, tumor tissues from a high-grade serous ovarian cancer patient were dissected into cubes and planted subcutaneously in female NOG mice.

Tumor volume was calculated as length × (square of width)/2. Elaiophylin, cisplatin, oxaliplatin, paclitaxel, and docetaxel were administered via intraperitoneal injections. Olaparib, talazoparib, and SHP099 were intragastrically administered. DMSO was used as a control.

### Statistical analysis

Statistical analyses were performed using GraphPad Prism software. Data are expressed as the mean ± SD or mean ± SEM, as indicated in the figure legends. The differences between the groups were analyzed using a Student’s two-tailed *t*-test. Statistical significance was set at *p* < 0.05.

## Supplementary information

Supplementary Materials

## Data Availability

The data and materials used in this study are available from the corresponding author upon reasonable request. The gene expression microarray data has been uploaded to GEO (GSE205529).
