# Supplementary material for: Elaiophylin triggers paraptosis and preferentially kills ovarian cancer drug-resistant cells by inducing MAPK hyperactivation
Source: Signal Transduct Target Ther. 2022 Sep 12;7:317. doi: 10.1038/s41392-022-01131-7 (PMC9468165; doi:10.1038/s41392-022-01131-7)
Supplement: Supplementary file 1 — Supplementary Materials [file 41392_2022_1131_MOESM1_ESM.docx]

Supplementary Materials for

**Elaiophylin triggers paraptosis and preferentially kills ovarian cancer drug-resistant cells by inducing MAPK hyperactivation**

Guan-Nan Li^1,2,†^, Xue-Jiao Zhao^1,2,†^, Zhen Wang^1,2,†^, Meng-Shi Luo^1,2^, Shen-Nan Shi^1,2^, Dan-Mei Yan^1,2^, Hua-Yi Li^1,2^, Jia-Hao Liu^1,2^, Yang Yang^1,3^, Jia-Hong Tan^1,2^, Ze-Yu Zhang^1,2^, Ru-Qi Chen^1,2^, Hui-Ling Lai^4^, Xiao-Yuan Huang^1,2^, Jian-Feng Zhou^1,3^, Ding Ma^1,2^, Yong Fang^1,2,^***** and Qing-Lei Gao^1,2,^*****

***Correspondence to:**

Qing-Lei Gao: [qingleigao@hotmail.com](mailto:qingleigao@hotmail.com)

Yong Fang: [tongjify@163.com](mailto:tongjify@163.com)

**This file includes:**

Supplementary Figures 1-21

Figure. S1.


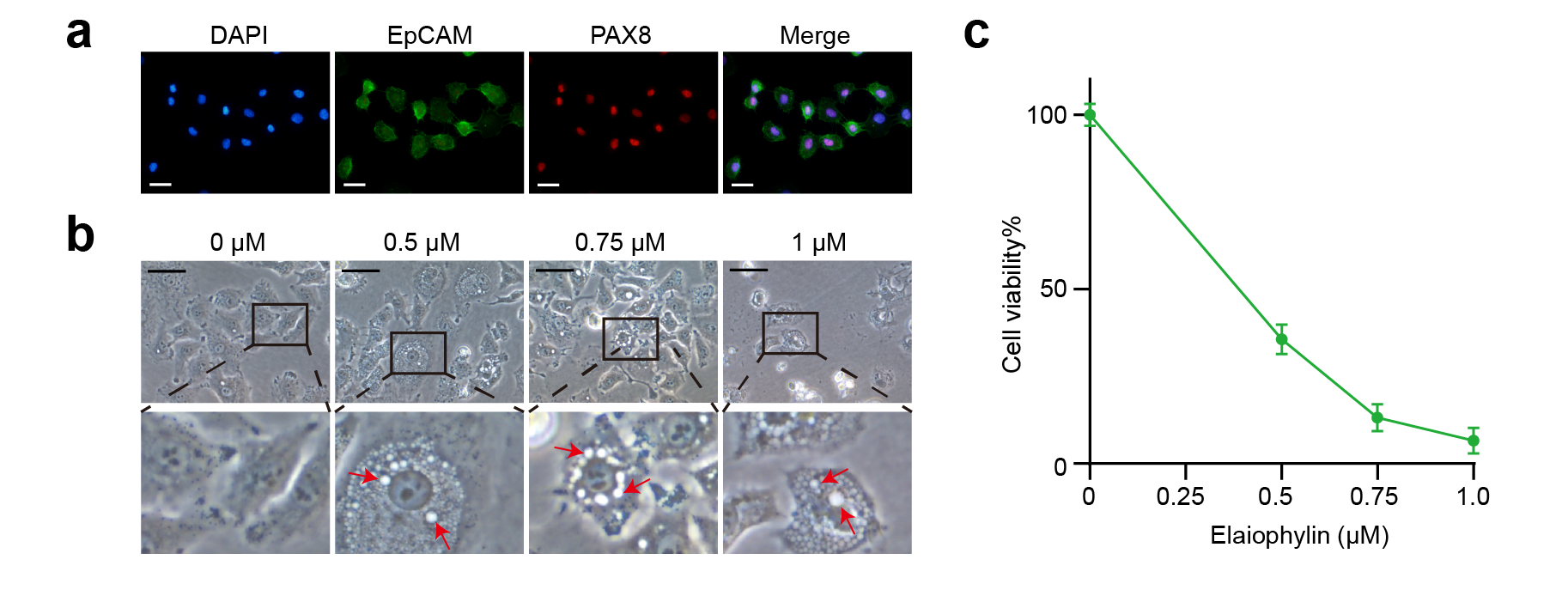


**Fig. S1** Elaiophylin induces cytoplasmic vacuoles in primary ovarian cancer cells.

**a** Primary ovarian cancer cells were isolated from the tumor tissues of a high-grade serous ovarian cancer patient, and stained using EpCAM antibody (green), PAX8 antibody (red), and DAPI (blue). Representative images are shown. Scale bar: 20 µm.

**b** Light microscopy images of primary ovarian cancer cells exposed to elaiophylin for 24 h. Arrows indicate cytoplasmic vacuoles. Scale bar: 50 µm.

**c** Cell viability of primary ovarian cancer cells exposed to elaiophylin for 24 h. Data are mean ± SD of three independent experiments.

Figure. S2.


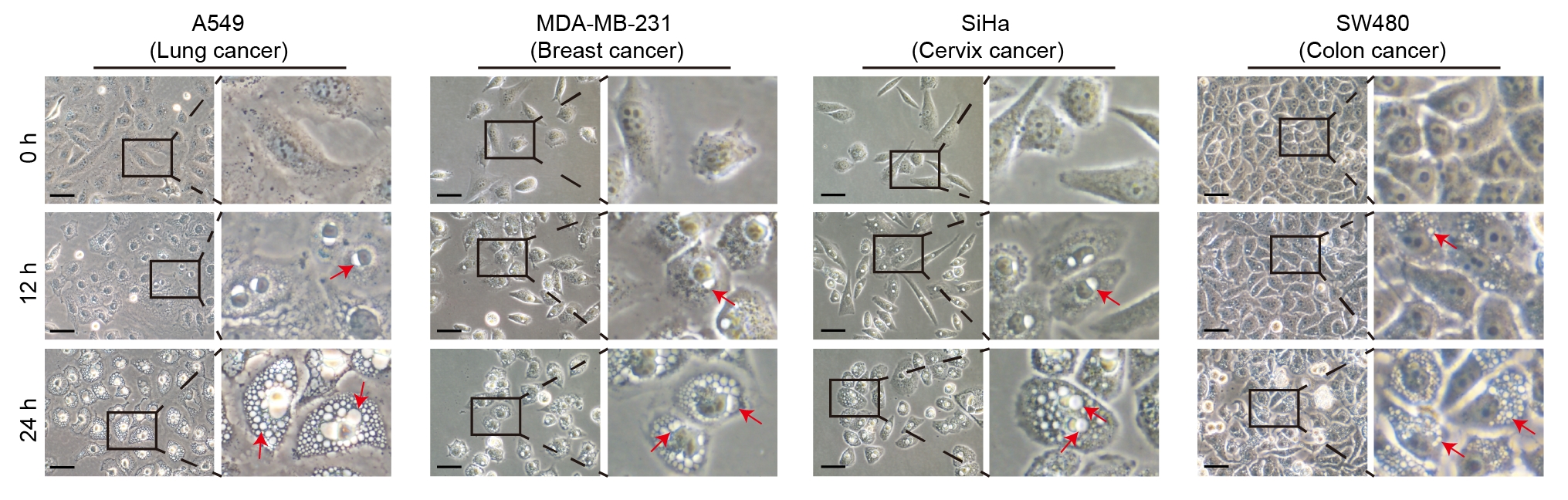


**Fig. S2** Elaiophylin induces cytoplasmic vacuoles in cell lines derived from other cancers.

A549, MDA-MB-231, SiHa, and SW480 cells were exposed to 0.5 µM elaiophylin. At the indicated time points, cells were observed by light microscopy. Arrows indicate cytoplasmic vacuoles. Scale bars: 50 µm.

Figure. S3.


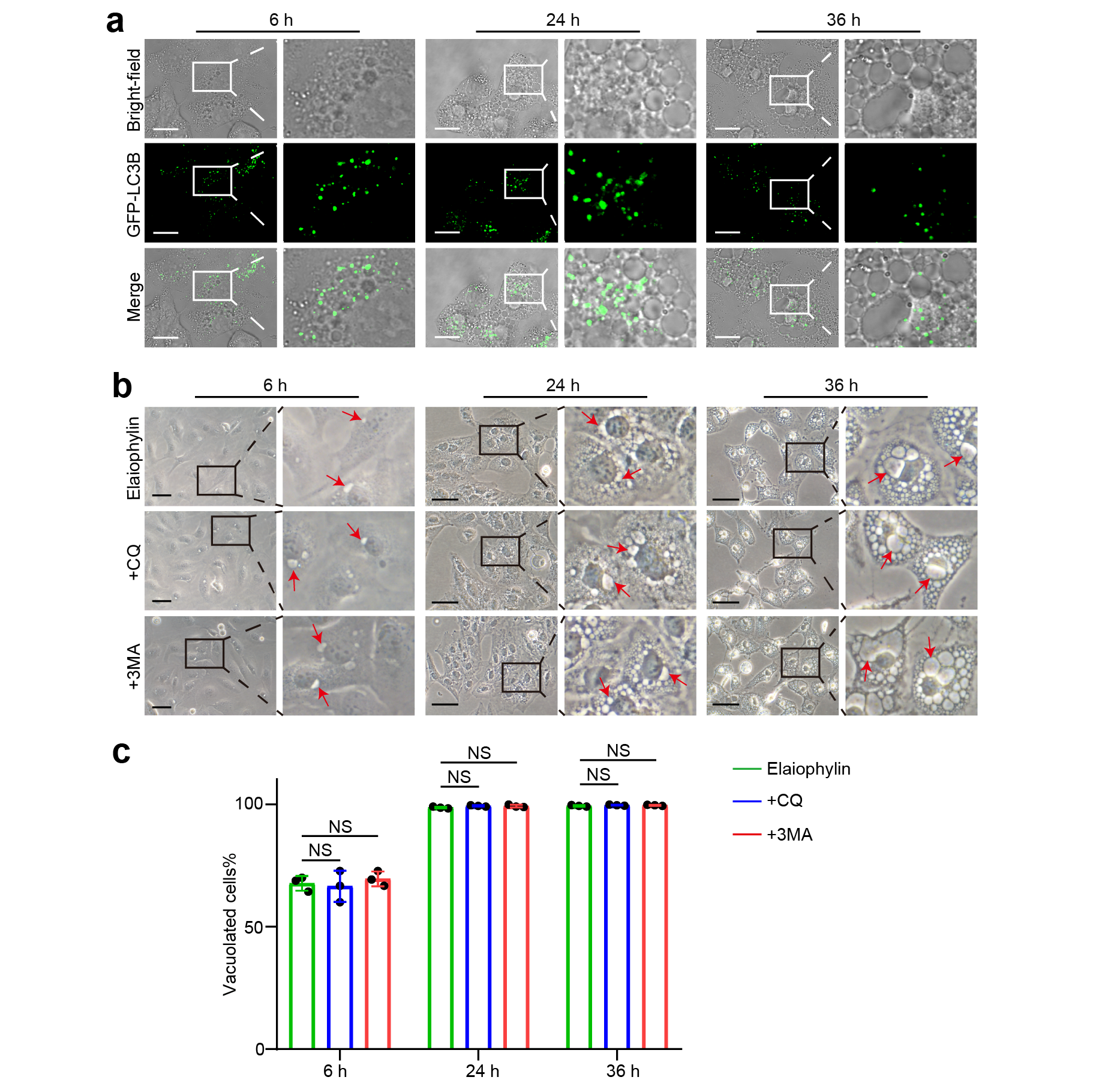


**Fig. S3** The relation between elaiophylin-induced cytoplasmic vacuoles and autophagosomes.

**a** SKOV3 cells expressing GFP-LC3B were exposed to 0.5 µM elaiophylin. At the indicated time points, cells were observed by confocal microscope. Representative bright-field and fluorescence images are shown. Scale bar: 20 µm.

**b** Representative light microscopy images of SKOV3 cells exposed to 0.5 µM elaiophylin alone, or in combination with CQ (25 µM) or 3-MA (5 mM). At the indicated time points, cells were observed by light microscopy. Arrows indicate cytoplasmic vacuoles. Scale bar: 50 µm.

**c** Quantification of data in (**b**), the proportion of cells displaying vacuoles was scored by visually examining at least 100 cells. Data are mean ± SD of three independent experiments (Two-tailed unpaired Student’s *t*-test, NS, *p* > 0.05).

Figure. S4.


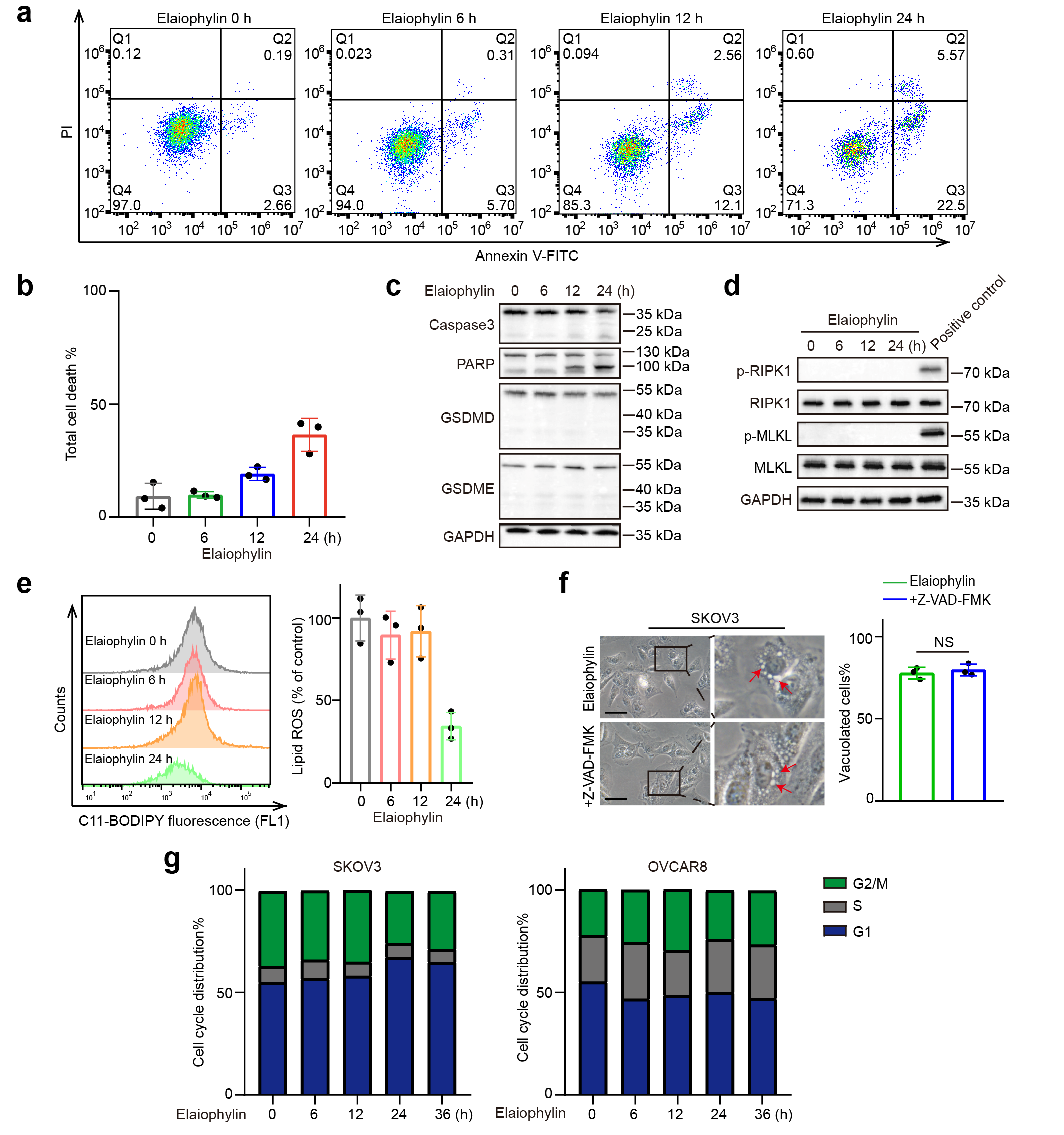


**Fig. S4** The possibilities in elaiophylin-induced cell death.

**a** SKOV3 cells were exposed to 0.5 µM elaiophylin. At the indicated time points, cells were collected and subject to FACS analysis of Annexin V and PI.

**b** Quantification of total cell death in (**a**). Data are mean ± SD of three independent experiments.

**c** Assessment of indicated protein levels using western blotting in SKOV3 cells exposed to 0.5 µM elaiophylin for indicated time points.

**d** Assessment of indicated protein levels using western blotting in SKOV3 cells exposed to 0.5 µM elaiophylin for indicated time points. HT-29 cells treated with TNFα, Smac mimetic, and Z-VAD-FMK were used as positive control.

**e** Left panel: FACS analysis of C11 BODIPY FL1 in SKOV3 cells exposed to 0.5 µM elaiophylin for indicated time points. Panel on the right is the quantification of the mean fluorescence intensity. Data are mean ± SD of three independent experiments.

**f** Left panel: light microscopy images of SKOV3 and OVCAR8 cells exposed to 0.5 µM elaiophylin alone or in combination with Z-VAD-FMK (25 µM) for 9 h. Arrows indicate cytoplasmic vacuoles. Scale bar: 50 µm. Right panel: proportion of cells displaying vacuoles was scored by visually examining at least 100 cells. Data are mean ± SD of three independent experiments (Two-tailed unpaired Student’s *t*-test, NS, *p* > 0.05).

**g** Cell cycle analysis of SKOV3 and OVCAR8 cells exposed to 0.5 µM elaiophylin for indicated time points.

Figure. S5.


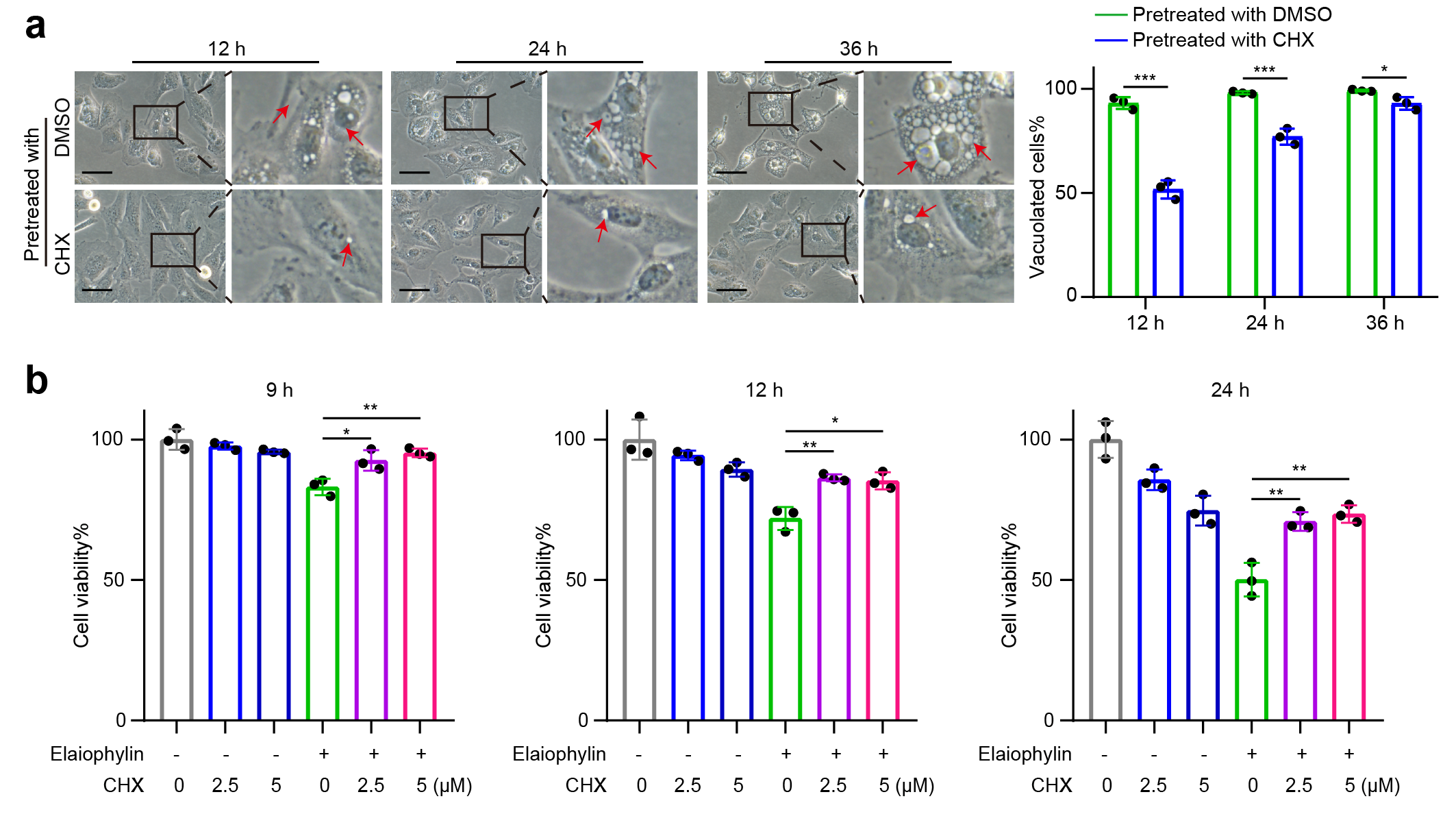


**Fig. S5** CHX inhibits elaiophylin-induced cytoplasmic vacuoles and cell death.

**a** Left panel: light microscopy images of SKOV3 cells exposed to 0.5 µM elaiophylin with pretreatment of DMSO or CHX (5 µM for 4 h). Arrows indicate cytoplasmic vacuoles. Scale bar: 50 µm. Right panel: proportion of cells displaying vacuoles was scored by visually examining at least 100 cells. Data are mean ± SD of three independent experiments (Two-tailed unpaired Student’s *t*-test, **p* < 0.05, ****p* < 0.001).

**b** Cell viability of SKOV3 cells exposed to 0.5 µM elaiophylin with pretreatment of DMSO or CHX (4 h). Data are mean ± SD of three independent experiments (Two-tailed unpaired Student’s *t*-test, **p* < 0.05, ** *p* < 0.01).

Figure. S6.


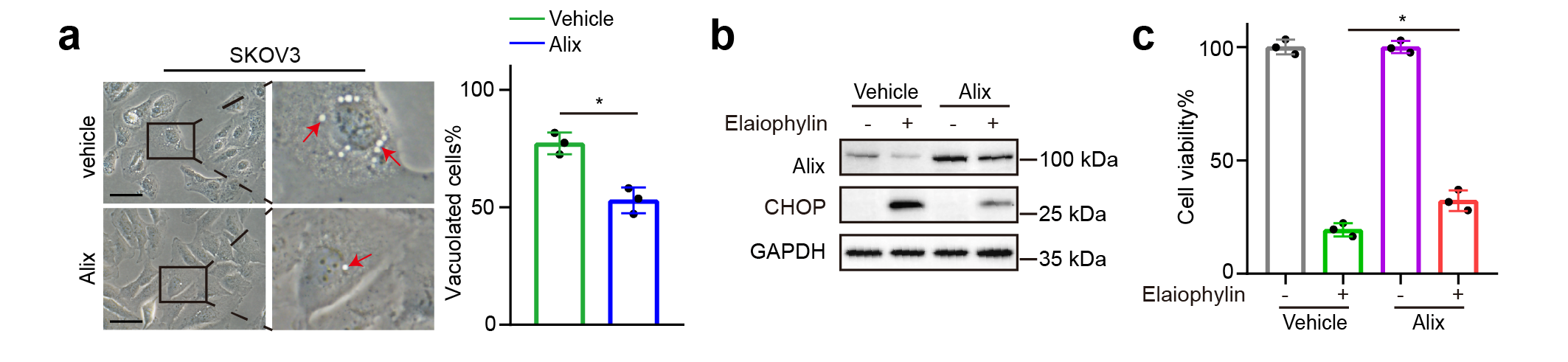


**Fig. S6** Overexpression of Alix inhibits elaiophylin-induced cytoplasmic vacuoles and cell death.

**a** Left panel: light microscopy images of SKOV3 cells overexpressing Alix exposed to 0.5 µM elaiophylin for 9 h. Arrows indicate cytoplasmic vacuoles. Scale bar: 50 µm. Right panel: proportion of cells displaying vacuoles was scored by visually examining at least 100 cells. Data are mean ± SD of three independent experiments (Two-tailed unpaired Student’s *t*-test, **p* < 0.05).

**b** Assessment of indicated protein levels using western blotting in SKOV3 cells overexpressing Alix exposed to 0.5 µM elaiophylin for 9 h.

**c** Cell viability of SKOV3 cells overexpressing Alix exposed to 0.5 µM elaiophylin for 36 h. Data are mean ± SD of three independent experiments (Two-tailed unpaired Student’s *t*-test, **p* < 0.05).

Figure. S7.


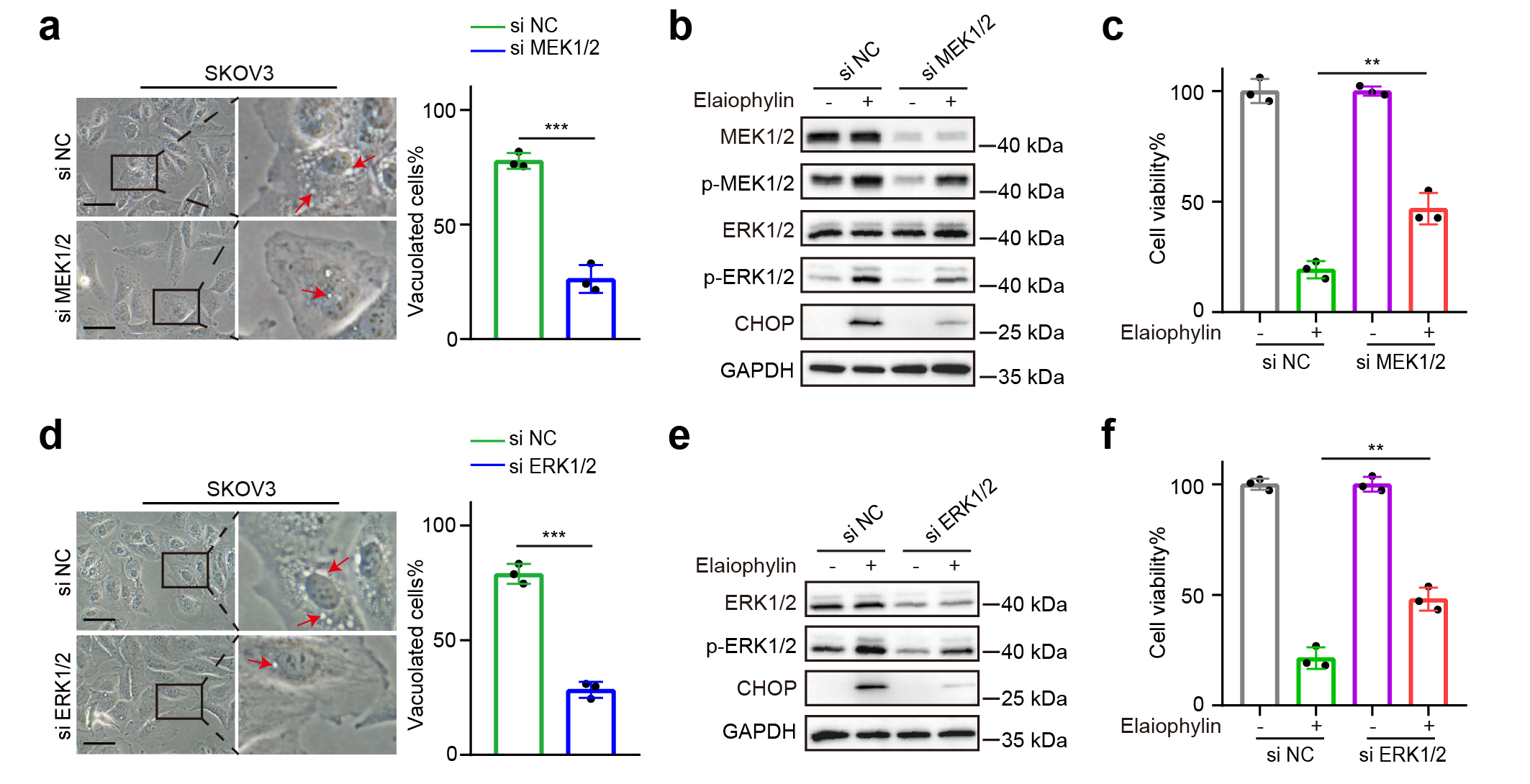


**Fig. S7** MEK1/2 knockdown or ERK1/2 knockdown attenuats elaiophylin-induced paraptosis.

**a** Left panel: light microscopy images of SKOV3 cells exposed to 0.5 µM elaiophylin for 9 h after MEK1/2 knockdown. Arrows indicate cytoplasmic vacuoles. Scale bar: 50 µm. Right panel: proportion of cells displaying vacuoles was scored by visually examining at least 100 cells. Data are mean ± SD of three independent experiments (Two-tailed unpaired Student’s *t*-test, ****p* < 0.001).

**b** Assessment of indicated protein levels using western blotting in SKOV3 cells exposed to 0.5 µM elaiophylin for 9 h after MEK1/2 knockdown.

**c** Cell viability of SKOV3 cells treated with 0.5 µM elaiophylin for 36 h after MEK1/2 knockdown. Data are mean ± SD of three independent experiments (Two-tailed unpaired Student’s *t*-test, ***p* < 0.01).

**d** Left panel: light microscopy images of SKOV3 cells treated with 0.5 µM elaiophylin for 9 h after ERK1/2 knockdown. Arrows indicate cytoplasmic vacuoles. Scale bar: 50 µm. Right panel: proportion of cells displaying vacuoles was scored by visually examining at least 100 cells. Data are mean ± SD of three independent experiments (Two-tailed unpaired Student’s *t*-test, ****p* < 0.001).

**e** Assessment of indicated protein levels using western blotting in SKOV3 cells exposed to 0.5 µM elaiophylin for 9 h after ERK1/2 knockdown.

**f** Cell viability of SKOV3 cells treated with 0.5 µM elaiophylin for 36 h after ERK1/2 knockdown. Data are mean ± SD of three independent experiments (Two-tailed unpaired Student’s *t*-test, ***p* < 0.01).

Figure. S8.


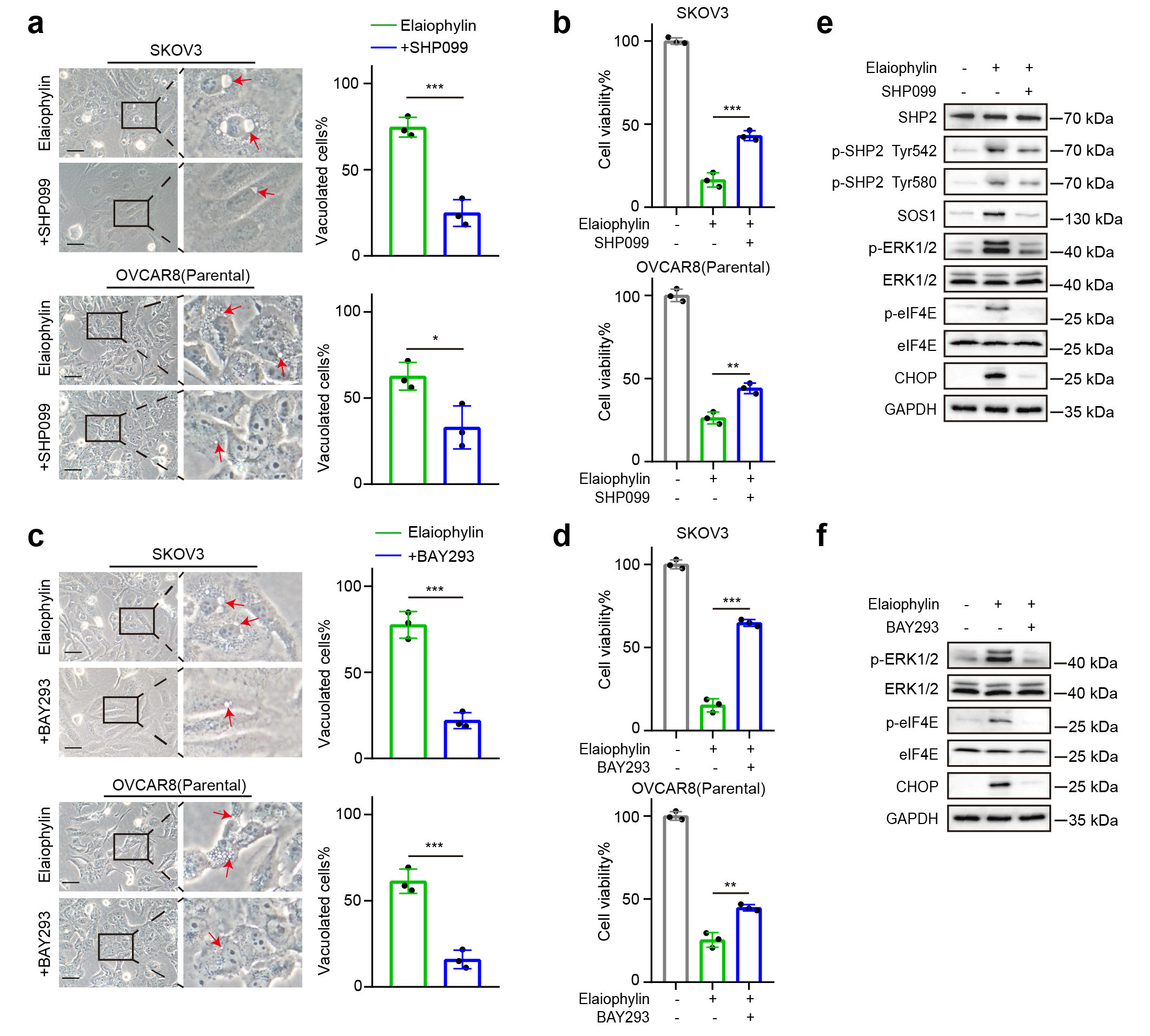


**Fig. S8** The activation of the SHP2/SOS1/MAPK pathway is required for elaiophylin-induced paraptosis.

**a** Left panel: representative light microscopy images of SKOV3 and OVCAR8 (parental) cells exposed to 0.5 µM elaiophylin alone or in combination of SHP099 (30 µM) for 9 h. Arrows indicate cytoplasmic vacuoles. Scale bar: 50 µm. Right panel: proportion of cells displaying vacuoles was scored by visually examining at least 100 cells. Data are mean ± SD of three independent experiments (Two-tailed unpaired Student’s *t*-test,**p* < 0.05, ****p* < 0.001).

**b** The viability of SKOV3 and OVCAR8 (parental) cells exposed to 0.5 µM elaiophylin alone or in combination of SHP099 (30 µM) for 36 h. Data are mean ± SD of three independent experiments (Two-tailed unpaired Student’s *t*-test, ***p* < 0.01, ****p* < 0.001).

**c** Left panel: representative light microscopy images of SKOV3 and OVCAR8 (parental) cells exposed to 0.5 µM elaiophylin alone or in combination of BAY293 (5 µM) for 9 h. Arrows indicate cytoplasmic vacuoles. Scale bar: 50 µm. Right panel: proportion of cells displaying vacuoles was scored by visually examining at least 100 cells. Data are mean ± SD of three independent experiments (Two-tailed unpaired Student’s *t*-test, ****p* < 0.001).

**d** The viability of SKOV3 and OVCAR8 (parental) cells exposed to 0.5 µM elaiophylin alone or in combination of BAY293 (5 µM) for 36 h. Data are mean ± SD of three independent experiments (Two-tailed unpaired Student’s *t*-test, ***p* < 0.01, ****p* < 0.001).

**e** Assessment of indicated protein levels using western blotting in SKOV3 cells exposed to 0.5 µM elaiophylin alone or in combination of SHP099 (30 µM) for 9 h.

**f** Assessment of indicated protein levels using western blotting in SKOV3 cells exposed to 0.5 µM elaiophylin alone or in combination of BAY293 (5 µM) for 9 h.

Figure. S9.


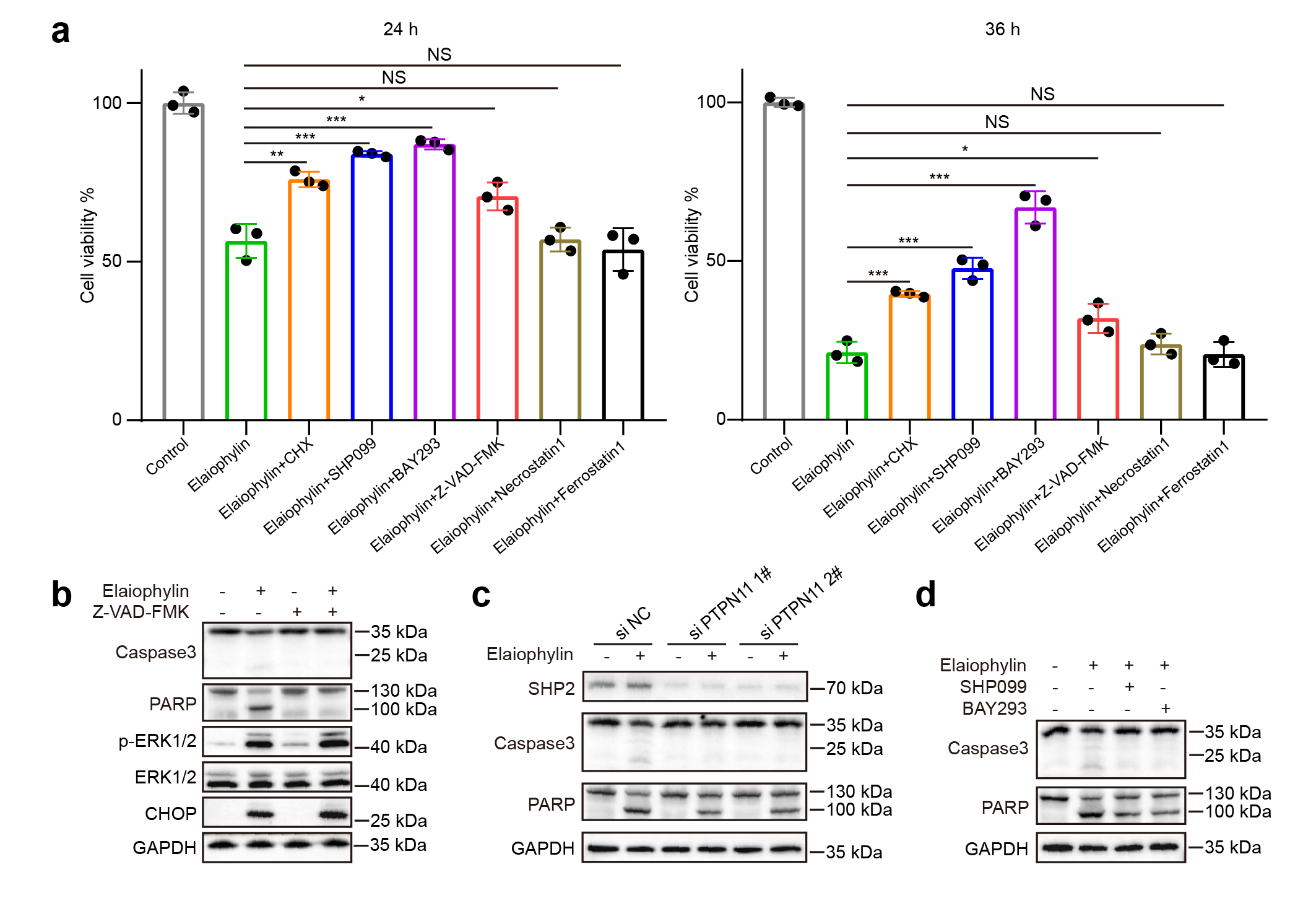


**Fig. S9** The relation between elaiophylin-induced paraptosis and apoptosis.

**a** The viability of SKOV3 cells exposed to 0.5 µM elaiophylin alone, or in combination of CHX (5 µM pretreatment for 4 h), SHP099 (30 µM), BAY293 (5 µM), Z-VAD-FMK (25 µM), Necrostatin1 (20 µM), Ferrostatin1 (10 µM), respectively. Data are mean ± SD of three independent experiments (Two-tailed unpaired Student’s *t*-test, ***p* < 0.01, ****p* < 0.001).

**b** Assessment of indicated protein levels using western blotting in SKOV3 cells exposed to 0.5 µM elaiophylin alone or in combination of Z-VAD-FMK (25 µM) for 24 h.

**c** Assessment of indicated protein levels using western blotting in SKOV3 cells exposed to 0.5 µM elaiophylin for 24 h after *PTPN11* knockdown.

**d** Assessment of indicated protein levels using western blotting in SKOV3 cells exposed to 0.5 µM elaiophylin alone, or in combination of SHP099 (30 µM) or BAY293 (5 µM) for 24 h.

Figure. S10.


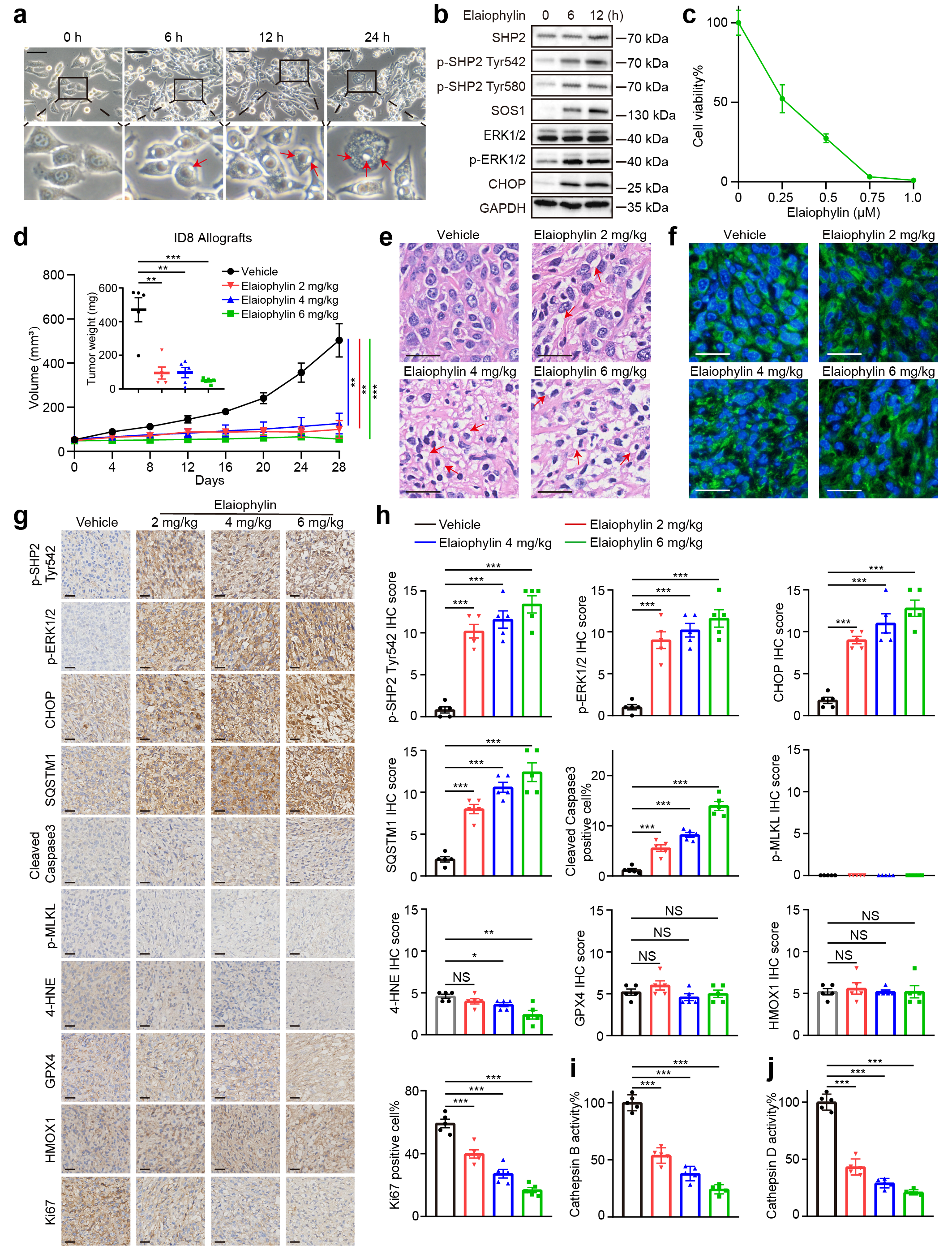


**Fig. S10** The effects of elaiophylin in murine ID8 models.

**a** ID8 cells were exposed to 0.5 µM elaiophylin. At the indicated time points, cells were observed by light microscopy. Arrows indicate cytoplasmic vacuoles. Scale bars: 50 µm.

**b** Assessment of indicated protein levels using western blotting in ID8 cells exposed to 0.5 µM elaiophylin.

**c** The viability of ID8 cells exposed to elaiophylin for 24 h. Data are mean ± SD of three independent experiments.

**d** Female C57BL-6J mice were inoculated subcutaneously with ID8 cells and were treated with vehicle or elaiophylin (2, 4, or 6 mg/kg/d) (n = 5 in each group). The tumor growth curves were determined by measuring the tumor volume at indicated time points after the beginning of treatment. Tumor weights were quantificated at the end of experiment. Data are mean ± SEM (Two-tailed unpaired Student’s *t*-test, ***p* < 0.01, ****p* < 0.001).

**e** Representative hematoxylin and eosin staining of tumor specimens in (**d**). Arrows indicate cytoplasmic vacuolation. Scale bar: 25 µm.

**f** Tumor specimens in (**d**) were stained using calnexin antibody (green) and DAPI (blue). Representative sections are shown. Scale bar: 25 µm.

**g** Representative images of immunohistochemical staining with indicated antibodies in tumor specimens from (**d**). Scale bar: 25 µm.

**h** Quantification of immunohistochemical scores in (**g**). Five sections were assessed per group and the mean of four randomly selected viewing fields were evaluated for every section. Data are mean ± SEM (Two-tailed unpaired Student’s *t*-test, NS, *p* > 0.05, ****p* < 0.001).

**i**, **j** Enzymatic activity of CTSB (**i**) and CTSD (**j**) in tumor tissues from (**d**). The results are presented as percentages of the vehicle group. Data are mean ± SEM (Two-tailed unpaired Student’s *t*-test, n = 5, ****p* < 0.001).

Figure. S11.


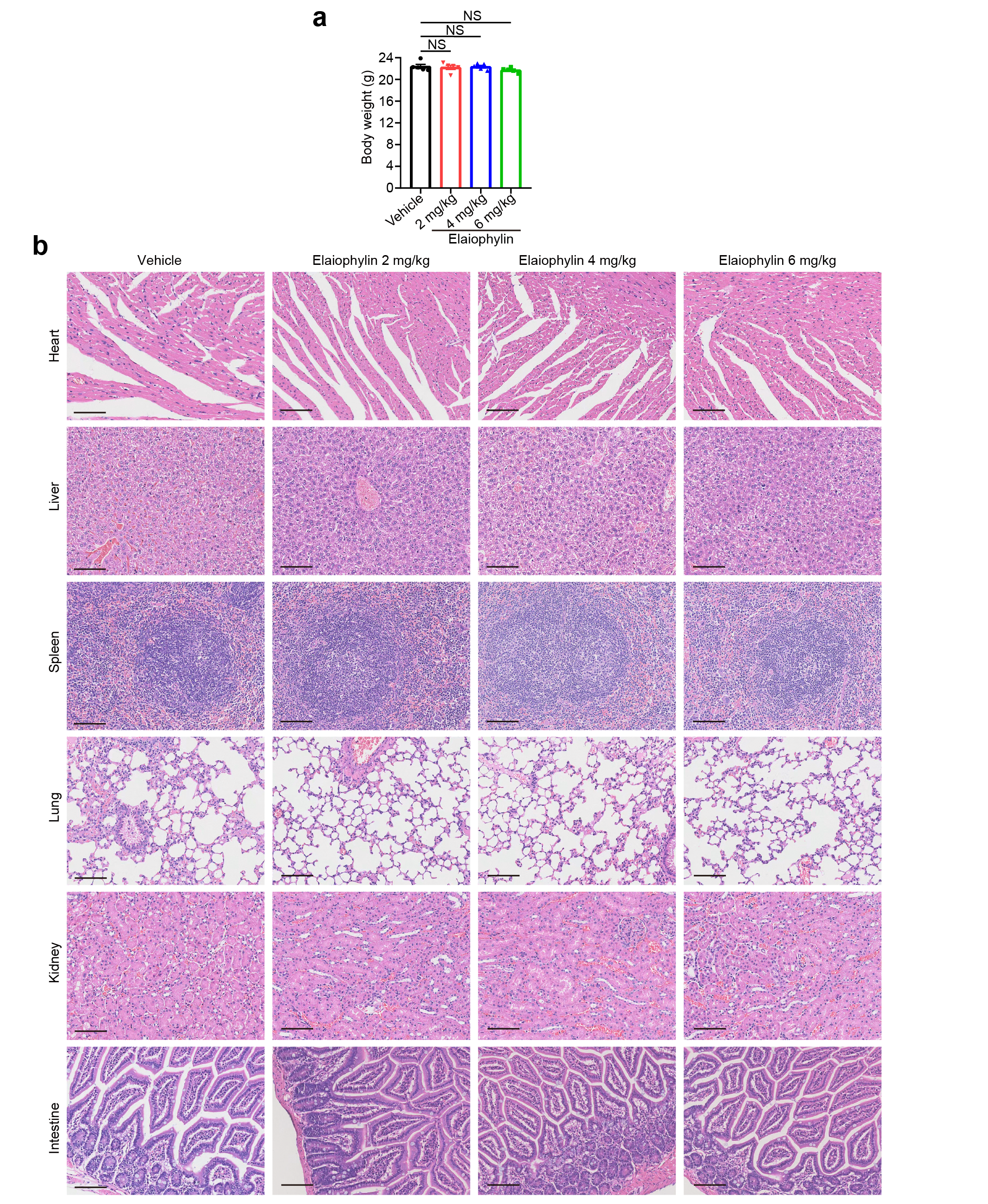


**Fig. S11** The safety of elaiophylin in ID8 allograft-bearing C57BL-6J mice.

**a** Quantification of body weight of mice at the end of the experiment. Data are mean ± SEM (Two-tailed unpaired Student’s *t*-test, n = 5, NS, *p* > 0.05).

**b** Representative hematoxylin and eosin staining of heart, liver, spleen, lung, kidney, and intestine tissue sections from the mice in each group. Scale bar: 100 µm.

Figure. S12.


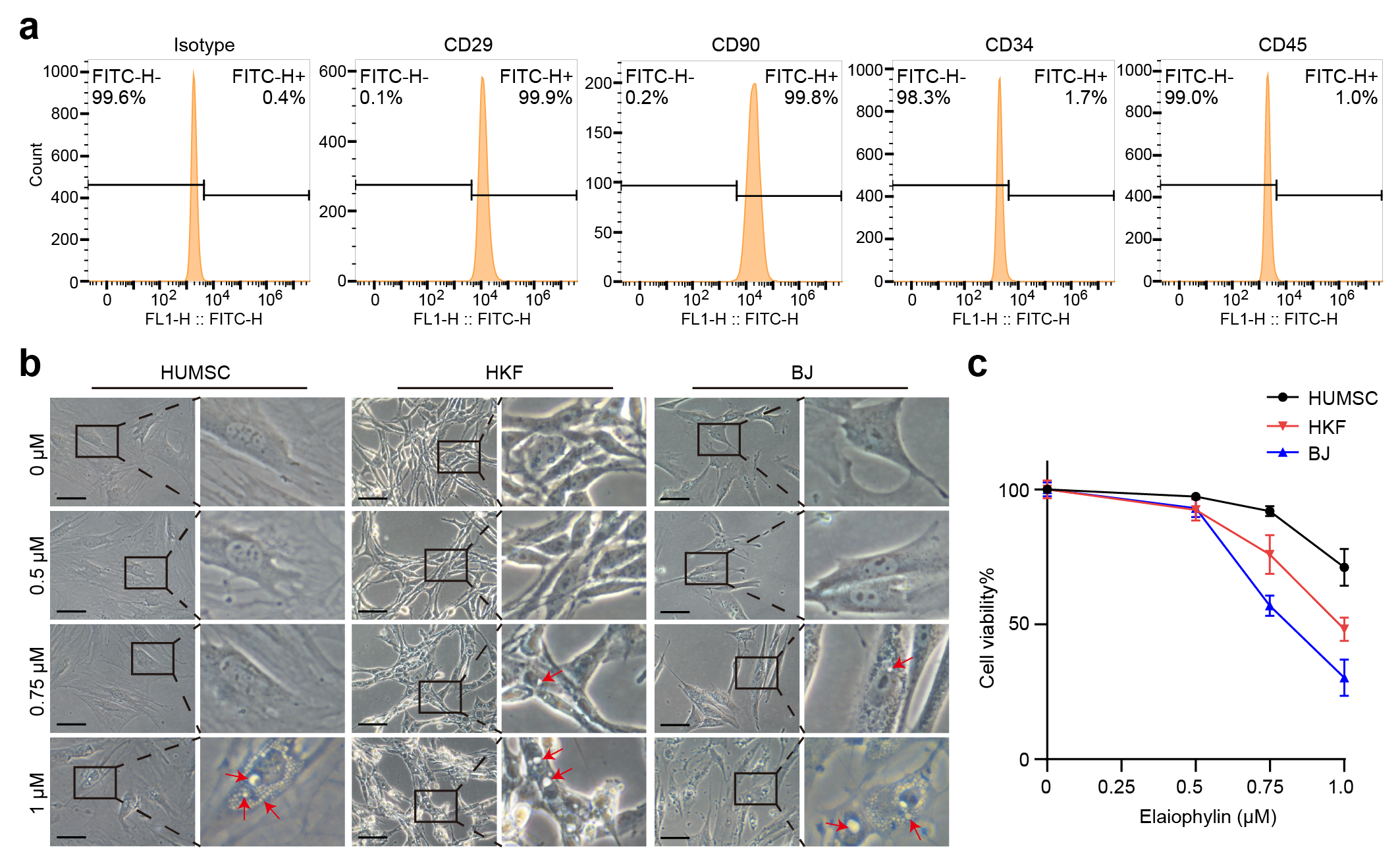


**Fig. S12** The effects of elaiophylin in nonneoplastic cells.

**a** HUMSC (human umbilical mesenchymal stem cells) were isolated, stained with antibodies against CD29, CD90, CD34, CD45, and subjected to FACS analysis for phenotype identification.

**b** Light microscopy images of HUMSC, HKF (human kidney fibroblast), and BJ (human fibroblast) cells exposed to elaiophylin for 24 h. Arrows indicate cytoplasmic vacuoles. Scale bar: 50 µm.

**c** The viability of HUMSC, HKF, and BJ cells exposed to elaiophylin for 24 h. Data are mean ± SD of three independent experiments.

Figure. S13.


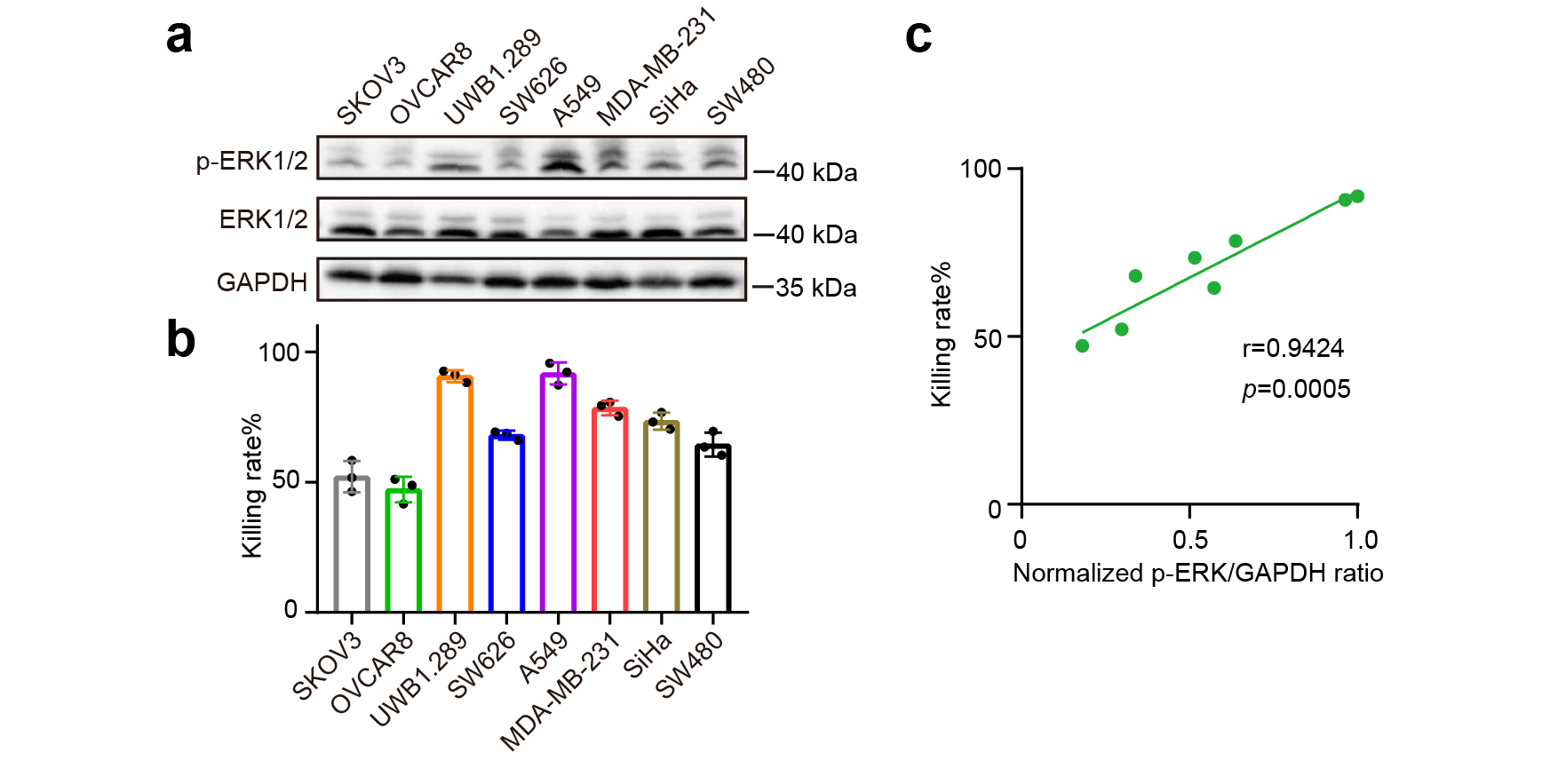


**Fig. S13** The correlation between the basal levels of MAPK and elaiophylin-induced cell death.

**a** Assessment of indicated protein levels using western blotting in indicated cells.

**b** Indicated cells were exposed to 0.5 µM elaiophylin for 24 h. The killing rates were estimated by CCK8. Data are mean ± SD of three independent experiments.

**c** Correlation analysis between normalized p-ERK/GAPDH ratios in (**a**) and killing rates in (**b**).

Figure. S14.


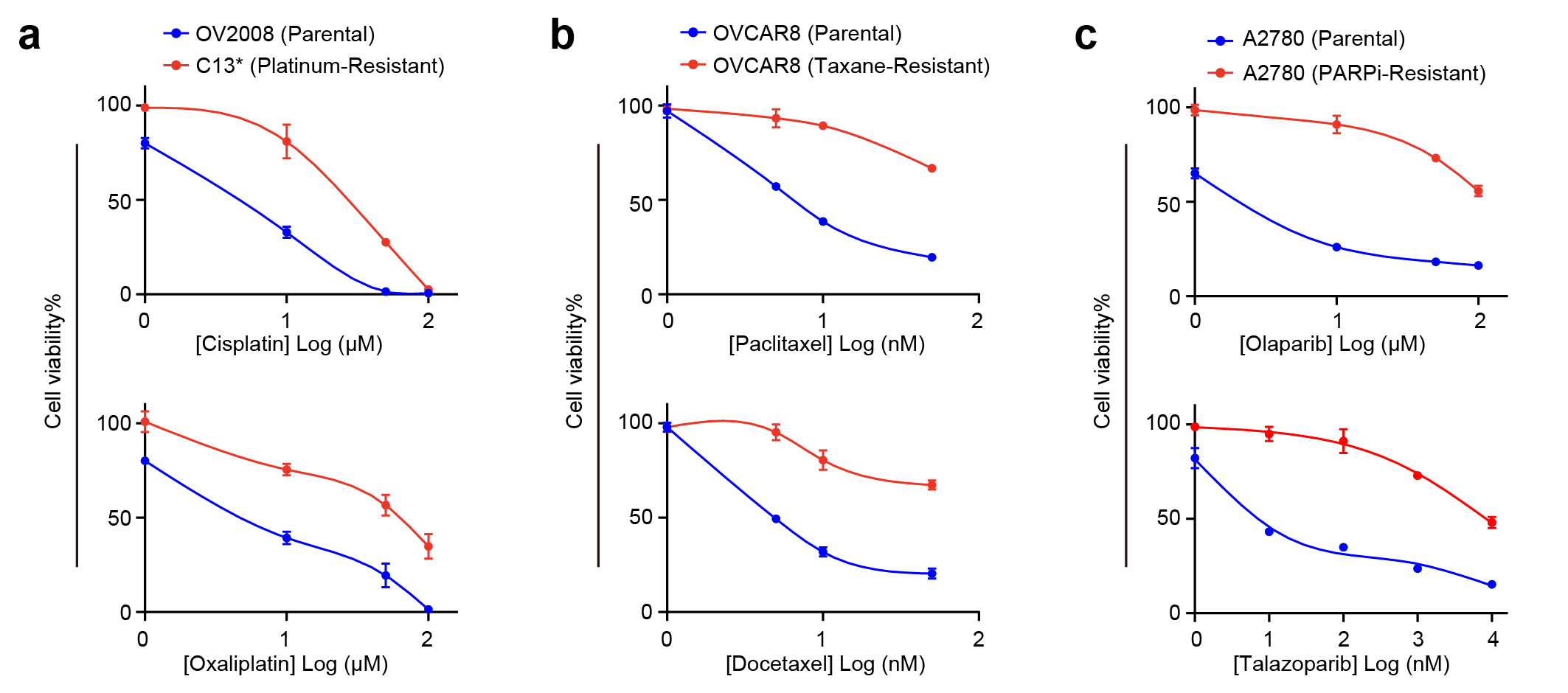


**Fig. S14** The sensitivity of parental and resistant ovarian cancer cells to platinum, taxane or PARPi.

**a** The viability of OV2008 and C13* cells treated with cisplatin or oxaliplatin for 48 h. The results are presented as percentages of the control. Data are mean ± SD of three independent experiments.

**b** The viability of parental OVCAR8 cells and taxane-resistant derivative treated with paclitaxel or docetaxel for 72 h. The results are presented as percentages of the control. Data are mean ± SD of three independent experiments.

**c** The viability of parental A2780 cells and PARPi-resistant derivative treated with olaparib or talazoparib for 72 h. The results are presented as percentages of the control. Data are mean ± SD of three independent experiments.

Figure. S15.


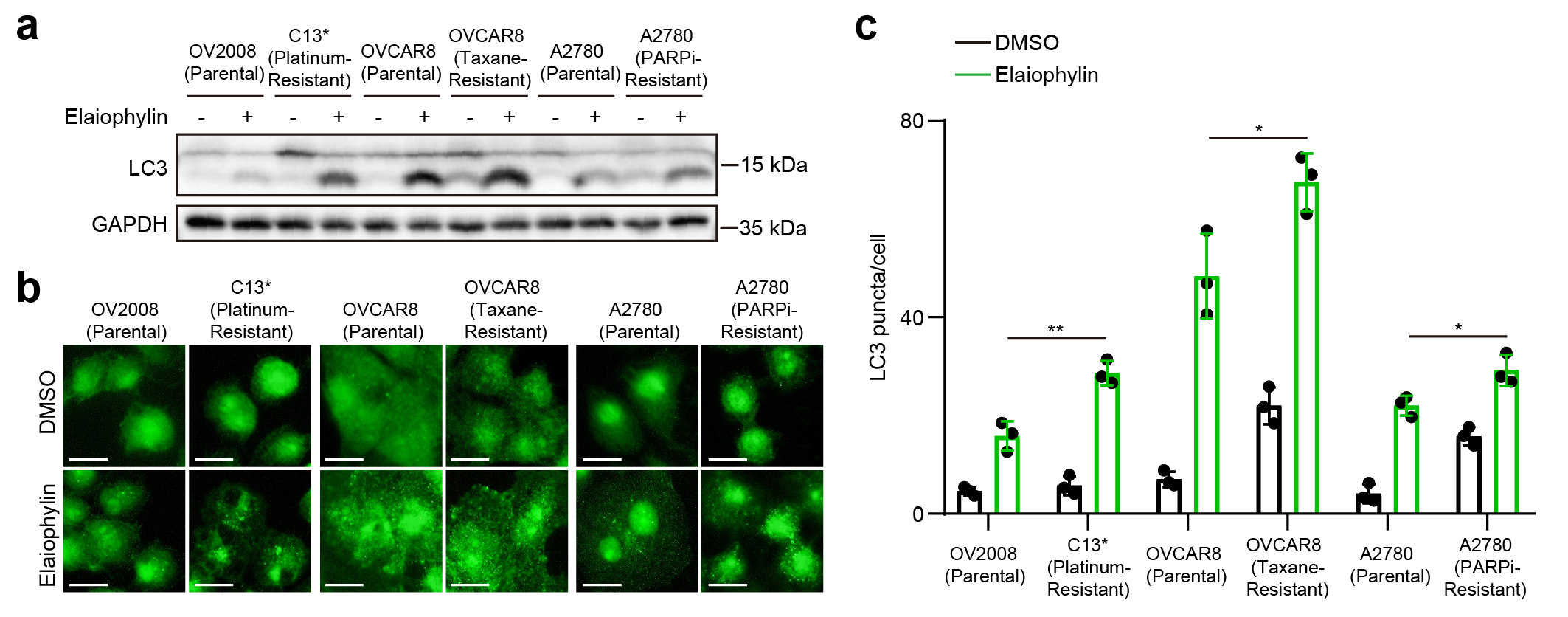


**Fig. S15** Elaiophylin induces stronger autophagy inhibition in drug-resistant cells.

**a** Assessment of indicated protein levels using western blotting in indicated cells exposed to 0.5 µM elaiophylin for 9 h.

**b** Indicated cells were exposed to 0.5 µM elaiophylin for 9 h and stained with LC3 antibody (green). Representative sections are shown. Scale bar: 10 µm.

**c** For every sample in (**b**)**,** LC3 puncta per cell were counted of at least 100 cells.. Data are mean ± SD of three independent experiments (Two-tailed unpaired Student’s *t*-test, **p* < 0.05, ***p* < 0.01).

Figure. S16.


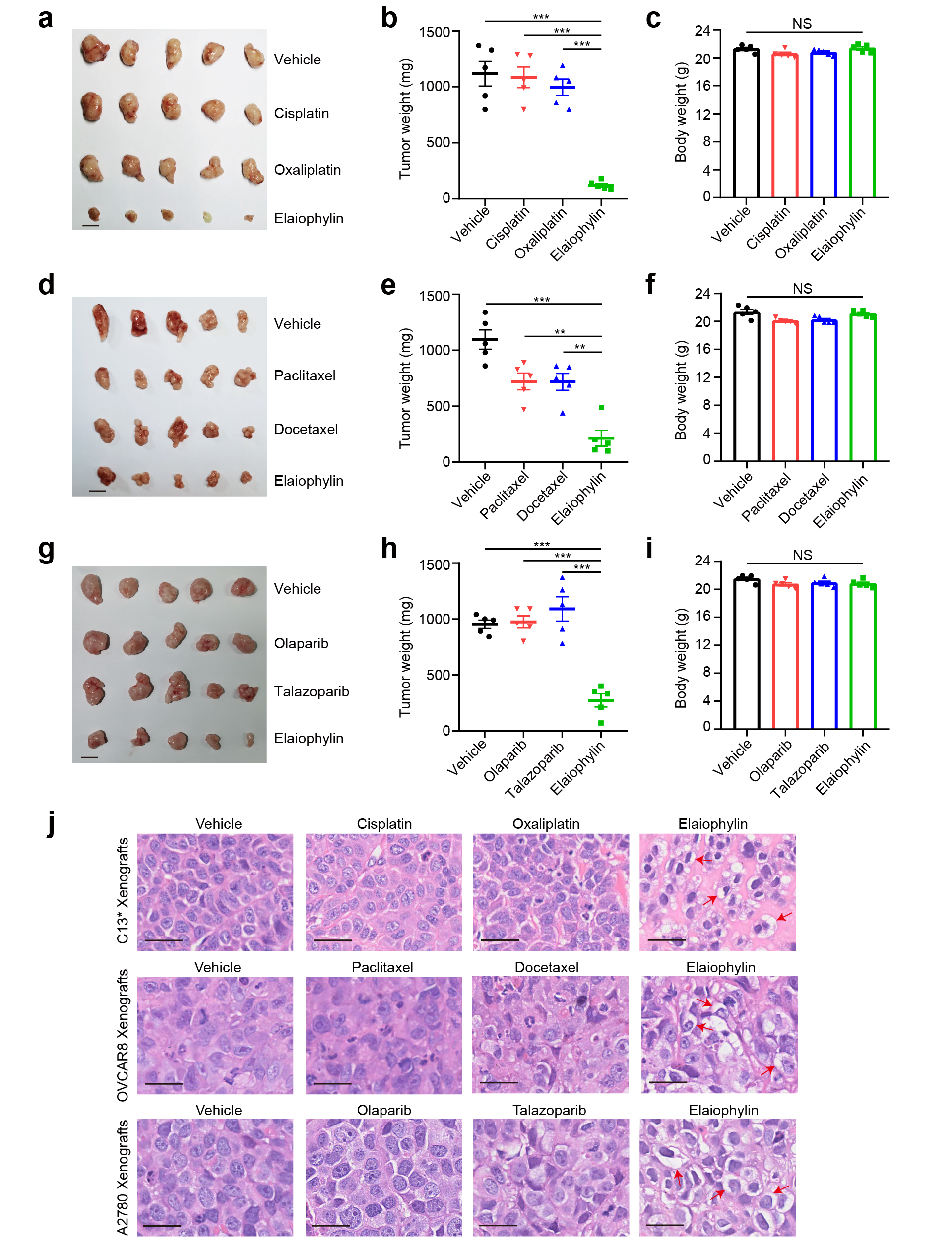


**Fig. S16** Elaiophylin exerts antitumor efficacy in drug-resistant models.

**a** Photograph of resected tumor tissues from C13* xenograft-bearing BALB/c mice. Scale bar: 1 cm.

**b** Quantification of tumor weight in (**a**). Data are mean ± SEM (Two-tailed unpaired Student’s *t*-test, n = 5, ****p* < 0.001).

**c** Quantification of body weight of C13* xenograft-bearing BALB/c mice at the end of experiment. Data are mean ± SEM (Two-tailed unpaired Student’s *t*-test, n = 5, NS, *p* > 0.05).

**d** Photograph of resected tumor tissues from OVCAR8 (taxane-resistant) xenograft-bearing BALB/c mice. Scale bar: 1 cm.

**e** Quantification of tumor weight in (**d**). Data are mean ± SEM (Two-tailed unpaired Student’s *t*-test, n = 5, ***p* < 0.01, ****p* < 0.001).

**f** Quantification of body weight of OVCAR8 (taxane-resistant) xenograft-bearing BALB/c mice at the end of experiment. Data are mean ± SEM (Two-tailed unpaired Student’s *t*-test, n = 5, NS, *p* > 0.05).

**g** Photograph of resected tumor tissues from A2780 (PARPi-resistant) xenograft-bearing BALB/c mice. Scale bar: 1 cm.

**h** Quantification of tumor weight in (**g**). Data are mean ± SEM (Two-tailed unpaired Student’s *t*-test, n = 5, ****p* < 0.001).

**i** Quantification of body weight of A2780 (PARPi-resistant) xenograft-bearing BALB/c mice at the end of experiment. Data are mean ± SEM (Two-tailed unpaired Student’s *t*-test, n = 5, NS, *p* > 0.05).

**j** Representative hematoxylin and eosin staining of tumor specimens in (**a**), (**d**), and (**g**). Arrows indicate cytoplasmic vacuolation. Scale bar: 25 µm.

Figure. S17.


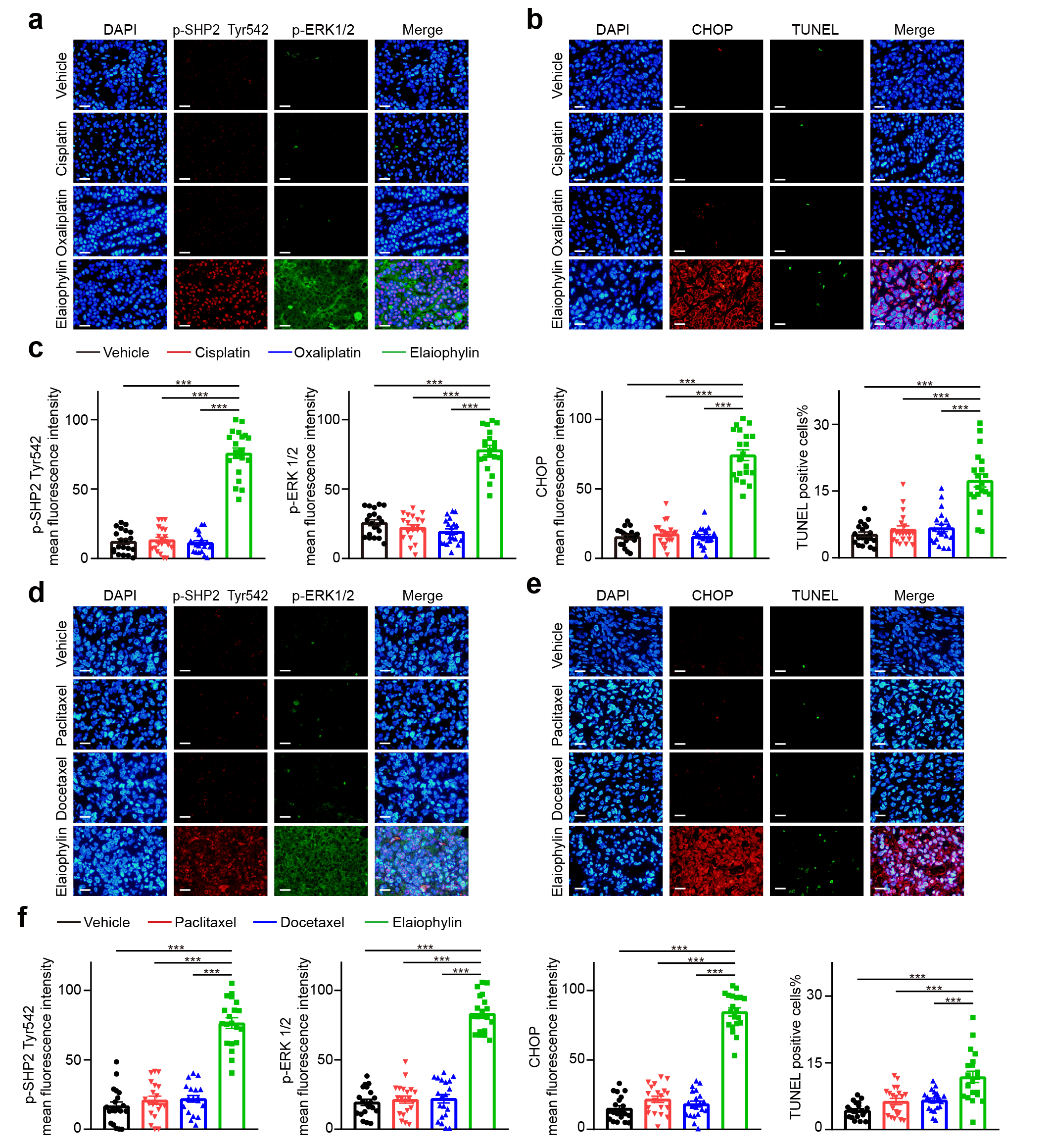


**Fig. S17** Elaiophylin induces paraptosis in chemo-resistant models.

**a** Sections of tumors from C13* xenograft-bearing BALB/c mice were stained using p-SHP2 Tyr542 antibody (red), p-ERK1/2 antibody (green), and DAPI (blue). Representative sections are shown. Scale bar: 20 µm.

**b** Sections of tumors from C13* xenograft-bearing BALB/c mice were stained using CHOP antibody (red), DAPI (blue), and using TUNEL assay (green). Representative sections are shown. Scale bar: 20 µm.

**c** Quantification of the mean fluorescence intensity and TUNEL positive proportion in (**a**) and (**b**). Four randomly selected viewing fields were individually evaluated per section and five sections were assessed per group. Data are mean ± SEM (Two-tailed unpaired Student’s *t*-test, ****p* < 0.001).

**d** Sections of tumors from OVCAR8 (taxane-resistant) xenograft-bearing BALB/c mice were stained using p-SHP2 Tyr542 antibody (red), p-ERK1/2 antibody (green), and DAPI (blue). Representative sections are shown. Scale bar: 20 µm.

**e** Sections of tumors from OVCAR8 (taxane-resistant) xenograft-bearing BALB/c mice were stained using CHOP antibody (red), DAPI (blue), and using TUNEL assay (green). Representative sections are shown. Scale bar: 20 µm.

**f** Quantification of the mean fluorescence intensity and TUNEL positive proportion in (**d**) and (**e**). Four randomly selected viewing fields were individually evaluated per section and five sections were assessed per group. Data are mean ± SEM (Two-tailed unpaired Student’s *t*-test, ****p* < 0.001).

Figure. S18.


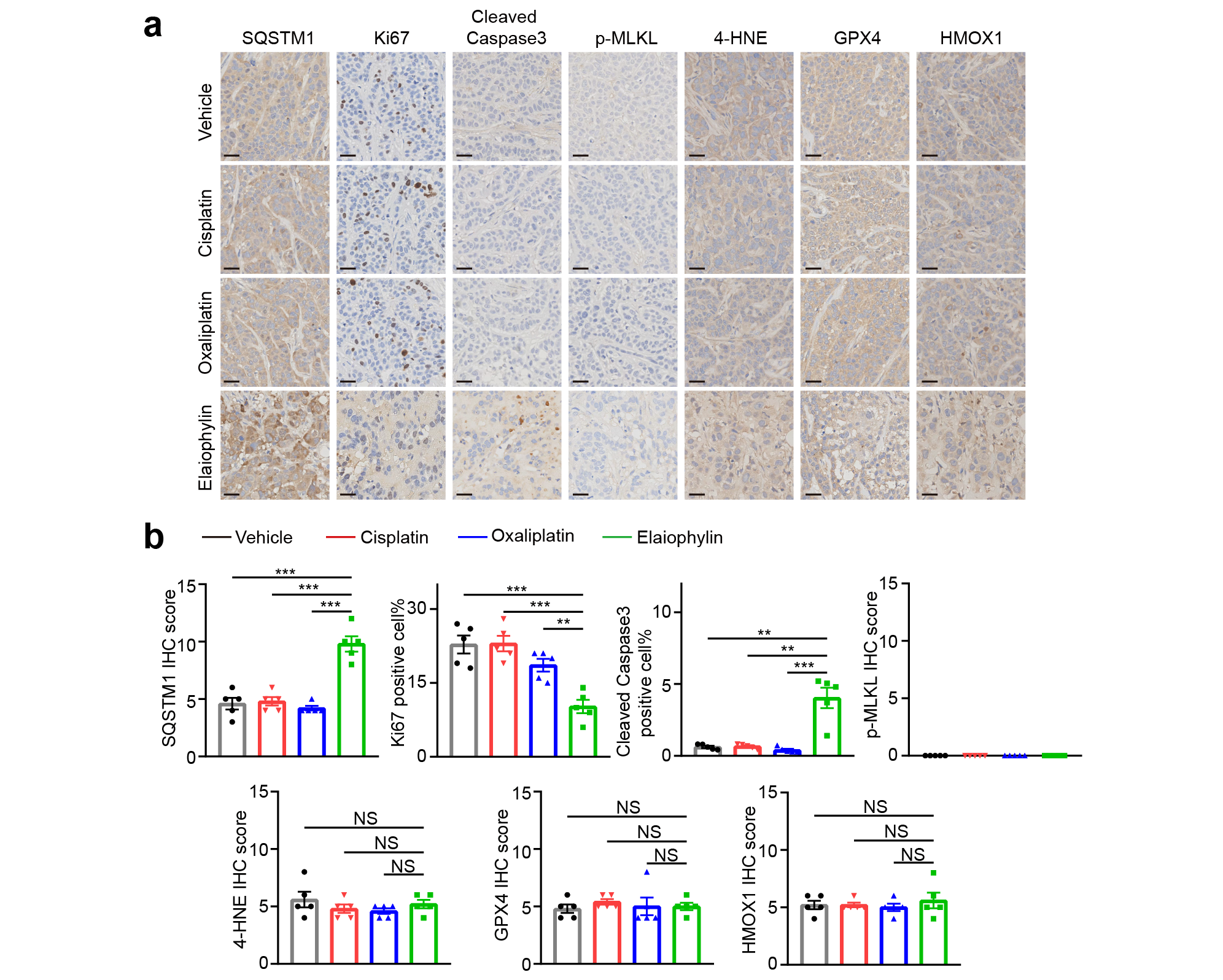


**Fig. S18** Elaiophylin induces autophagy inhibition, apoptosis, and proliferation inhibition in platinum-resistant model.

**a** Representative images of immunohistochemical staining with indicated antibodies in tumor specimens from C13* (platinum-resistant) xenograft-bearing BALB/c mice. Scale bar: 25 µm.

**b** Quantification of immunohistochemical scores in (**a**). Five sections were assessed per group and the mean of four randomly selected viewing fields were evaluated for every section. Data are mean ± SEM (Two-tailed unpaired Student’s *t*-test, NS, *p* > 0.05, ***p* < 0.01, ****p* < 0.001).

Figure. S19.


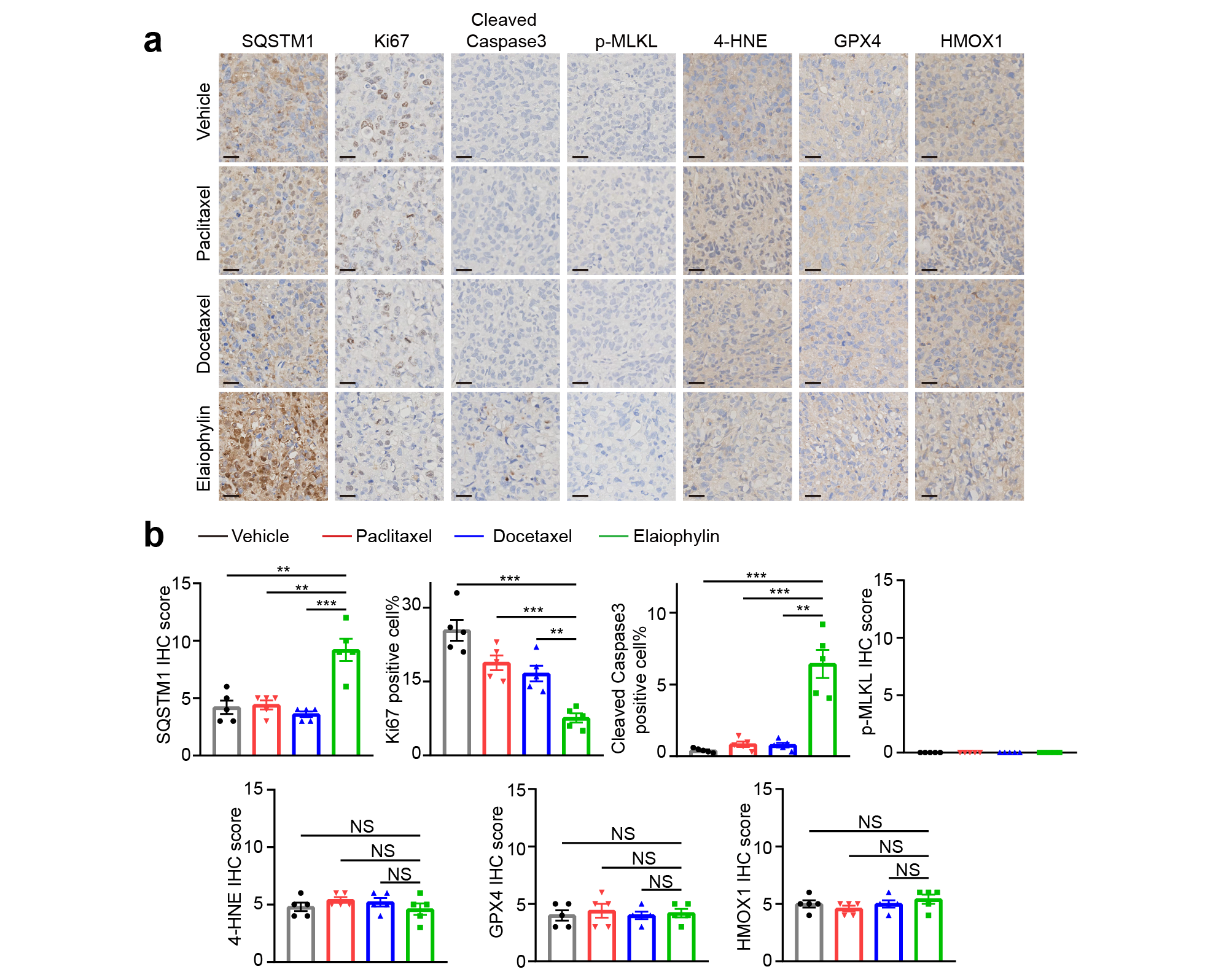


**Fig. S19** Elaiophylin induces autophagy inhibition, apoptosis, and proliferation inhibition in taxane-resistant model.

**a** Representative images of immunohistochemical staining with indicated antibodies in tumor specimens from OVCAR8 (taxane-resistant) xenograft-bearing BALB/c mice. Scale bar: 25 µm.

**b** Quantification of immunohistochemical scores in (**a**). Five sections were assessed per group and the mean of four randomly selected viewing fields were evaluated for every section. Data are mean ± SEM (Two-tailed unpaired Student’s *t*-test, NS, *p* > 0.05, ***p* < 0.01, ****p* < 0.001).

Figure. S20.


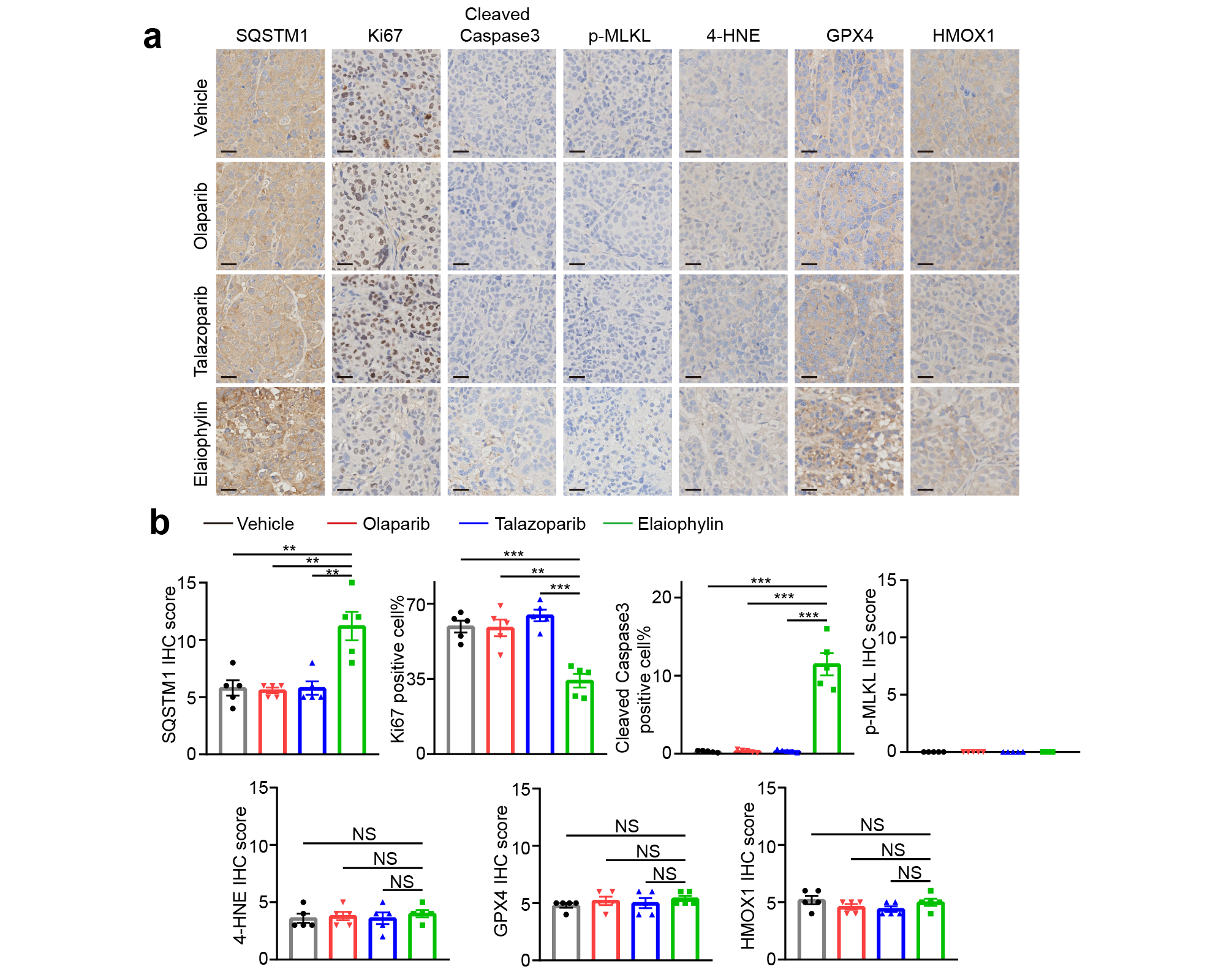


**Fig. S20** Elaiophylin induces autophagy inhibition, apoptosis, and proliferation inhibition in PARPi-resistant model.

**a** Representative images of immunohistochemical staining with indicated antibodies in tumor specimens from A2780 (PARPi-resistant) xenograft-bearing BALB/c mice. Scale bar: 25 µm.

**b** Quantification of immunohistochemical scores in (**a**). Five sections were assessed per group and the mean of four randomly selected viewing fields were evaluated for every section. Data are mean ± SEM (Two-tailed unpaired Student’s *t*-test, NS, *p* > 0.05, ***p* < 0.01, ****p* < 0.001).

Figure. S21.


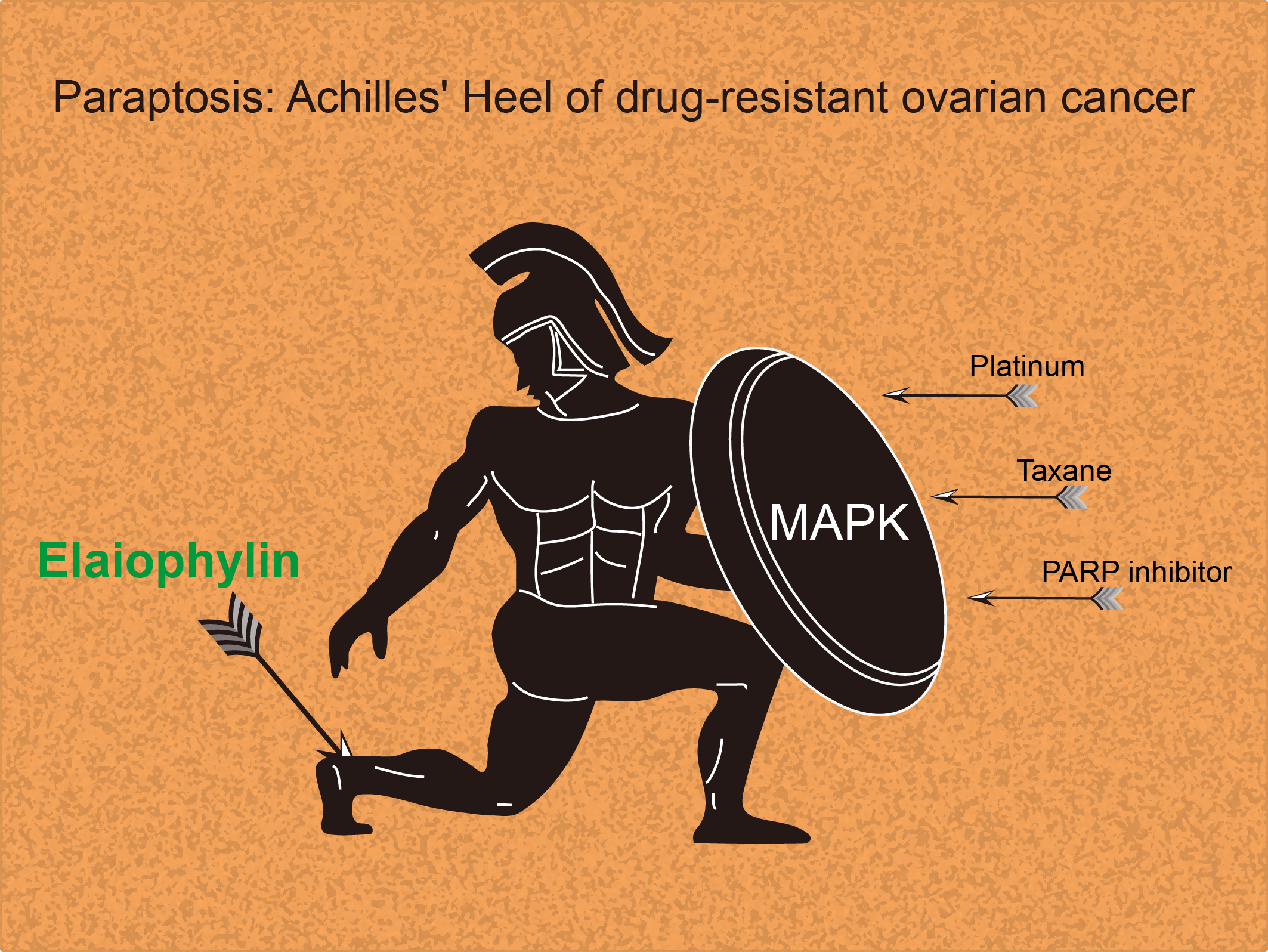


**Fig. S21** The Achilles’ Heel of drug-resistant ovarian cancer.

Achilles’ Heel is a weakness in spite of overall strength, which could lead to downfall. MAPK activation liberated ovarian cancer cells from the cytotoxicity of platinum, taxane or PARP inhibitors, but contemporaneously became a vulnerability to elaiophylin-induced paraptosis.
